# Supplementary material for: Bimetallic nickel-molybdenum/tungsten nanoalloys for high-efficiency hydrogen oxidation catalysis in alkaline electrolytes
Source: Nat Commun. 2020 Sep 22;11:4789. doi: 10.1038/s41467-020-18585-4 (PMC7508880; doi:10.1038/s41467-020-18585-4)
Supplement: Supplementary file 1 — Supplementary Information [file 41467_2020_18585_MOESM1_ESM.pdf]

Supplementary Information for

Bimetallic nickel-molybdenum/tungsten nanoalloys for high-efficiency hydrogen oxidation catalysis in alkaline electrolytes

Duan et al.

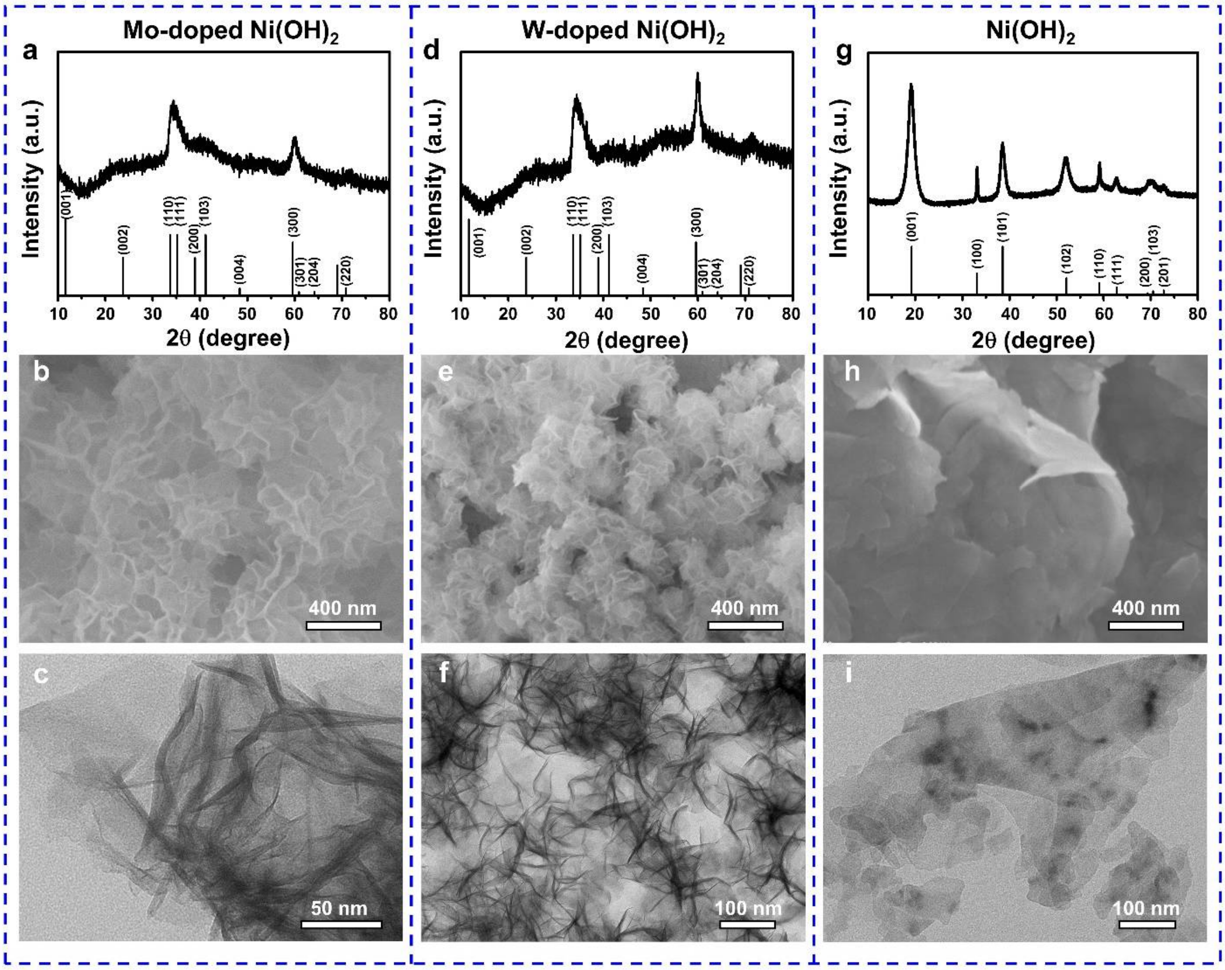

Supplementary Figure 1. Physical characterization of Mo-doped $Ni(OH)_2$, W-doped $Ni(OH)_2$ and $Ni(OH)_2$ precursors. a-c, XRD pattern (**a**), SEM image (**b**) and TEM image (**c**) of Mo-doped $Ni(OH)_2$ precursors. **d-f**, XRD pattern (**d**), SEM image (**e**) and TEM image (**f**) of W-doped $Ni(OH)_2$ precursors. **g-i**, XRD pattern (**g**), SEM image (**h**) and TEM image (**i**) of $Ni(OH)_2$ precursors. The inserted lines in **a** and **d** are indexed to $3Ni(OH)_2 \cdot 2H_2O$ (*JCPDS* 22-0444). The inserted lines in **i** are indexed to $Ni(OH)_2$ (*JCPDS* 14-0117). The phase-transition of $Ni(OH)_2$ after Mo (W) doping together with no obvious Mo (W) oxide diffraction peaks suggest the existence and homogeneous dispersion of Mo (W) atoms in the $Ni(OH)_2$ nanosheets.

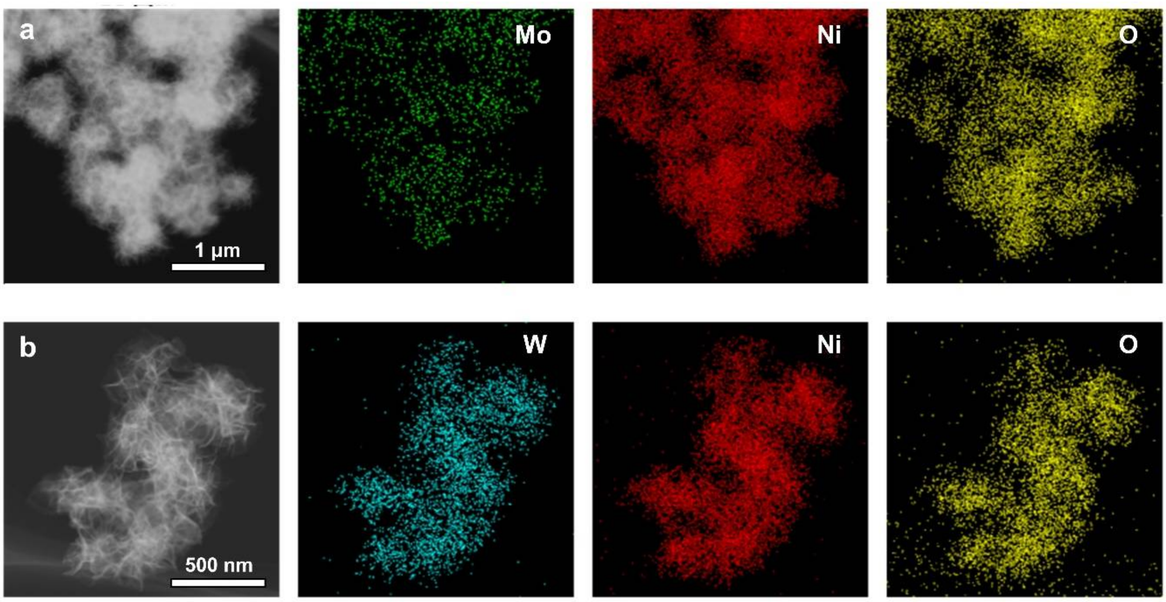

Supplementary Figure 2. STEM-EDX elemental mappings. a, Mo-doped $Ni(OH)_2$ precursors. **b,** W-doped $Ni(OH)_2$ precursors**.** STEM-EDX elemental mappings show the well distribution of Mo (W) and Ni elements throughout the precursors of Mo (W)-doped $Ni(OH)_2$ nanosheets, respectively, benefiting to form homogenous alloying structure during the following annealing process in H_2/Ar atmosphere.

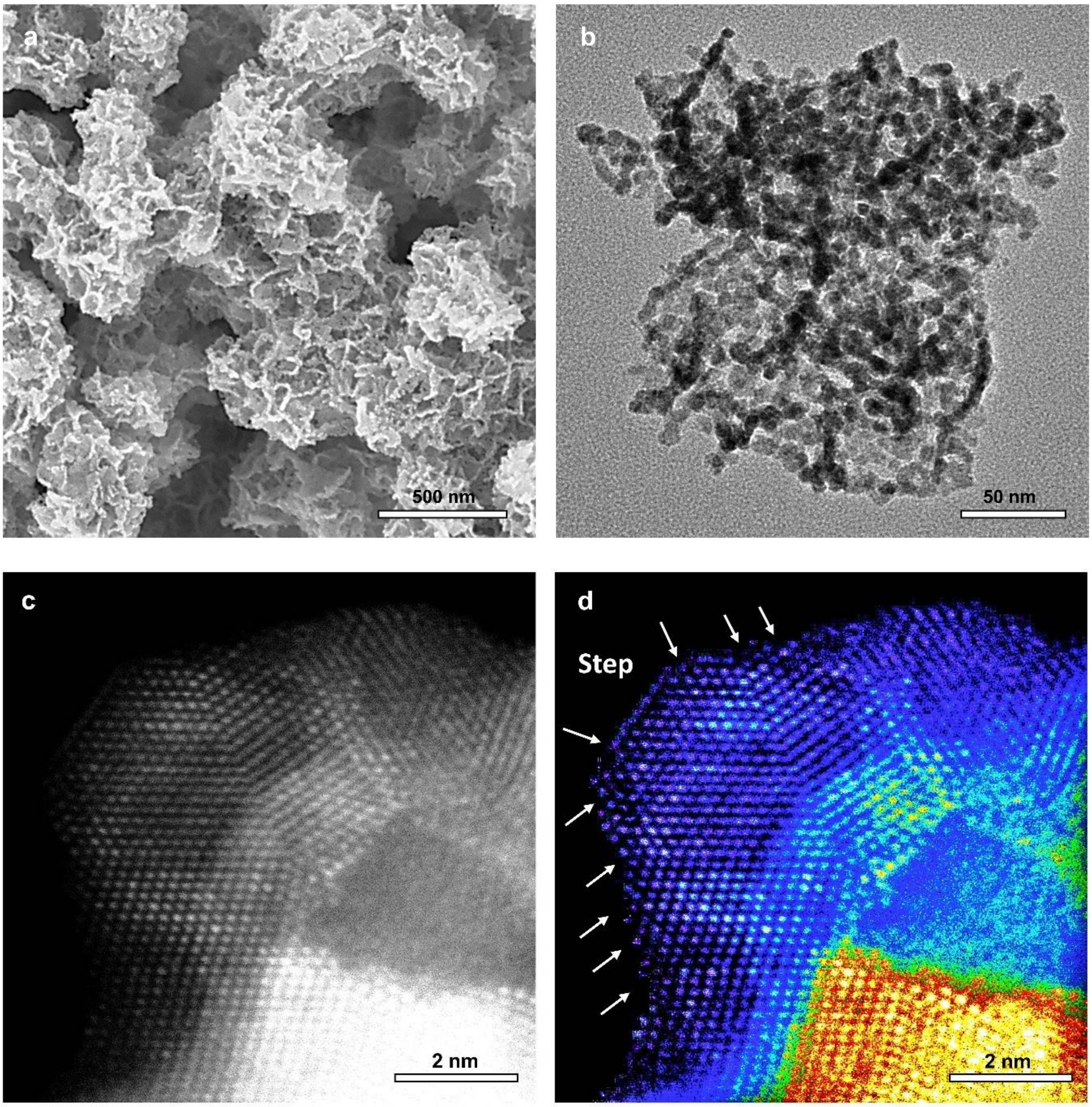

Supplementary Figure 3. Physical characterization of $MoNi_4$ alloy. a, SEM image. **b**, TEM image. The $MoNi_4$ alloy show sheet-like morphology comprised by interconnected nanoparticles (size: smaller than 10 nm), exhibiting its porous feature. **c**, HRTEM image. **d**, Colored HRTEM image transformed from **c**. It should be noted that the color in **d** is used to display the steps and edges of $MoNi_4$ nanoalloy rather than indicating atom kinds. Abundant steps and edges can been seen in **c** and **d**，demonstrating its low atomic coordination environment, increased active sites and surface areas.

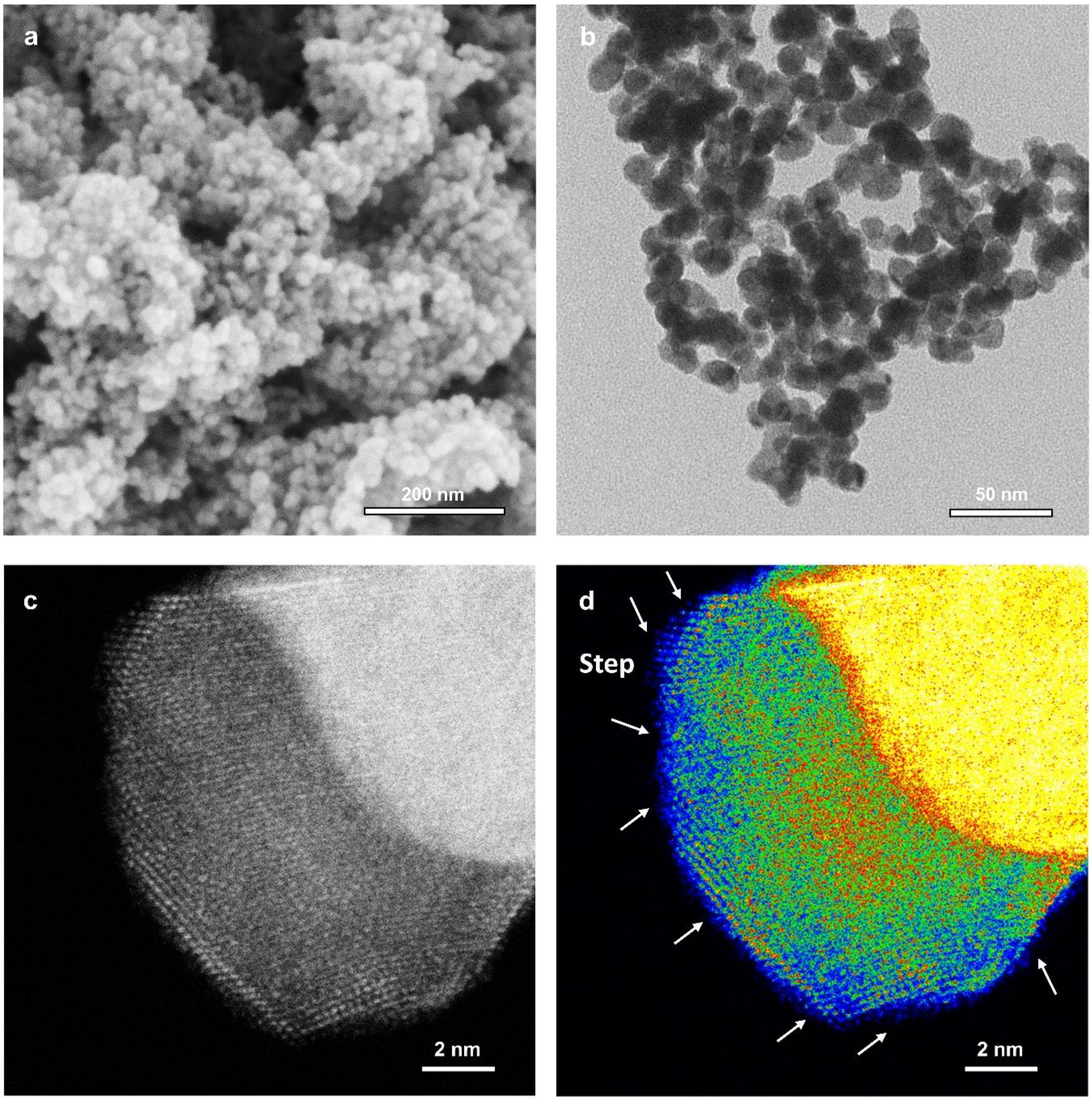

Supplementary Figure 4. Physical characterization of WNi_4 alloy. a, SEM image. **b**, TEM image. Overlapping nanoparticles with an average diameter of 18 nm can be observed in WNi_4 alloy due to higher reduction temperature in the synthetic process of WNi_4 alloy. **c**, HRTEM image. **d**, Colored HRTEM image transformed from **c**. It should be noted that the color in **d** is used to display the steps and edges of WNi_4 nanoalloy rather than indicating atom kinds. Abundant steps and edges can been seen in **c** and **d**.

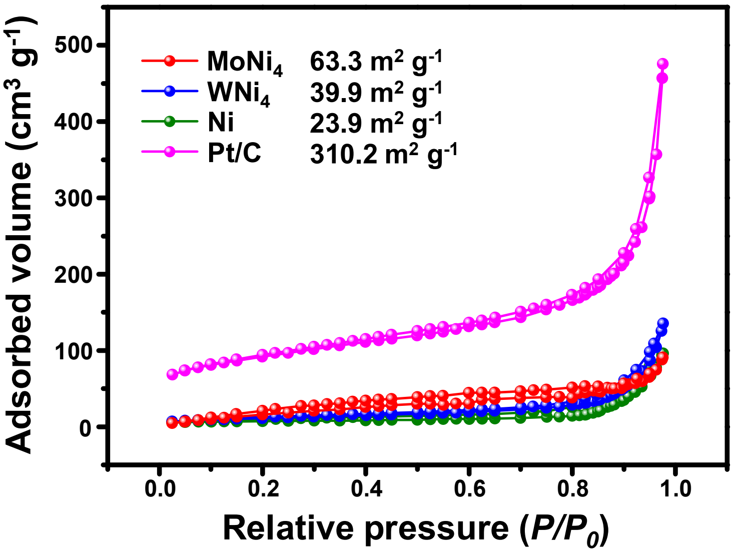

Supplementary Figure 5. | Brunauer–Emmett–Teller (BET) surface area analysis of $MoNi_4$, WNi_4, freshly-synthesized Ni and commercial Pt/C. The BET surface area of $MoNi_4$ alloy is 63.3 $m^2\ g^{-1}$, larger than the value of WNi_4 alloy (39.9 $m^2\ g^{-1}$) and Ni metal (23.9 $m^2\ g^{-1}$), promoting more active sites to be exposed in electrolyte. Meanwhile, the Pt/C is observed with lager surface area (310.2 $m^2\ g^{-1}$), suggesting the high quality of our selected commercial catalyst.

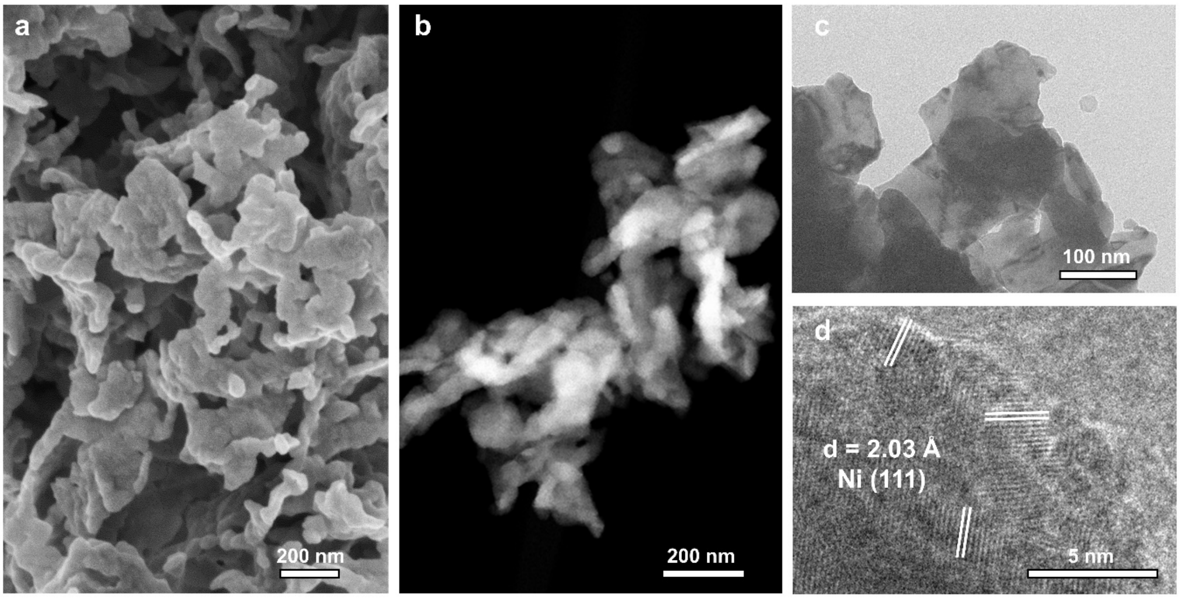

Supplementary Figure 6. Morphology characterization of freshly-synthesized Ni. a, SEM image. **b**, STEM image. **c**, TEM image. **d**, HRTEM image. The freshly-synthesized Ni obtains the sheet-like morphology form $Ni(OH)_2$ nanosheet precursors. However, its thickness becomes larger, leading to less surface area which has been confirmed by the BET results (Supplementary Fig. 5).

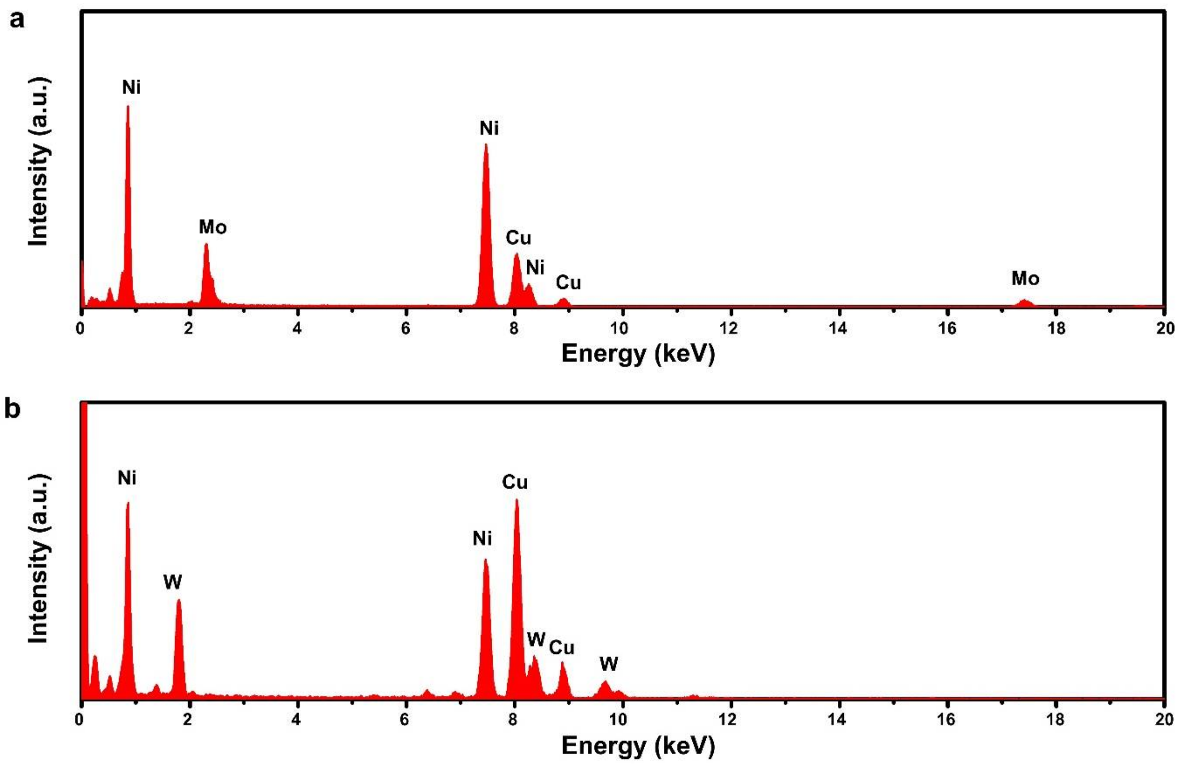

Supplementary Figure 7. EDX spectra. a, $MoNi_4$ alloy. **b,** WNi_4 alloy. Based on the EDX spectra, the Mo:Ni ratio for $MoNi_4$ alloy and W:Ni ratio for WNi_4 alloy are about 1:4.17 and 1:3.85, respectively, which are consistent with ICP-AES results (Supplementary Table 1).

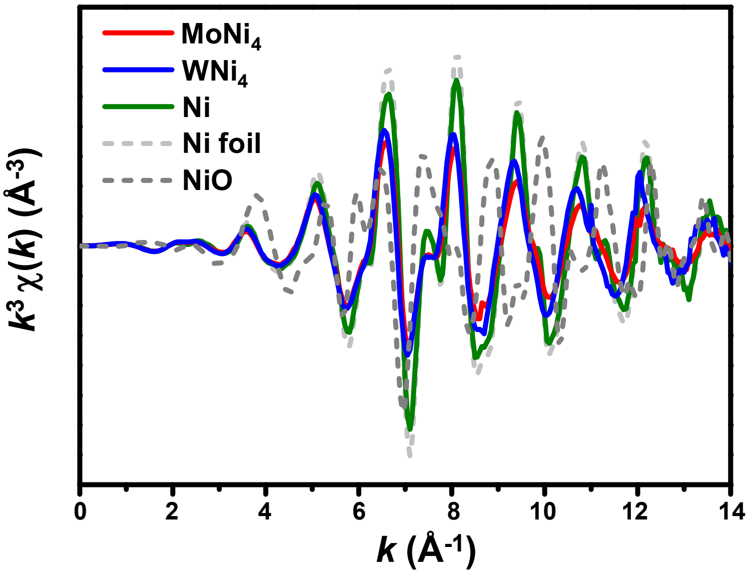

Supplementary Figure 8. Ni K-edge EXAFS oscillation spectra of $MoNi_4$, WNi_4, freshly-synthesized Ni, Ni foil and NiO. The EXAFS oscillation reveals the discrepancy of the coordination environment of Ni species in $MoNi_4$, WNi_4 and freshly-synthesized Ni compared with NiO, suggesting the metallic structure of the studied alloys.

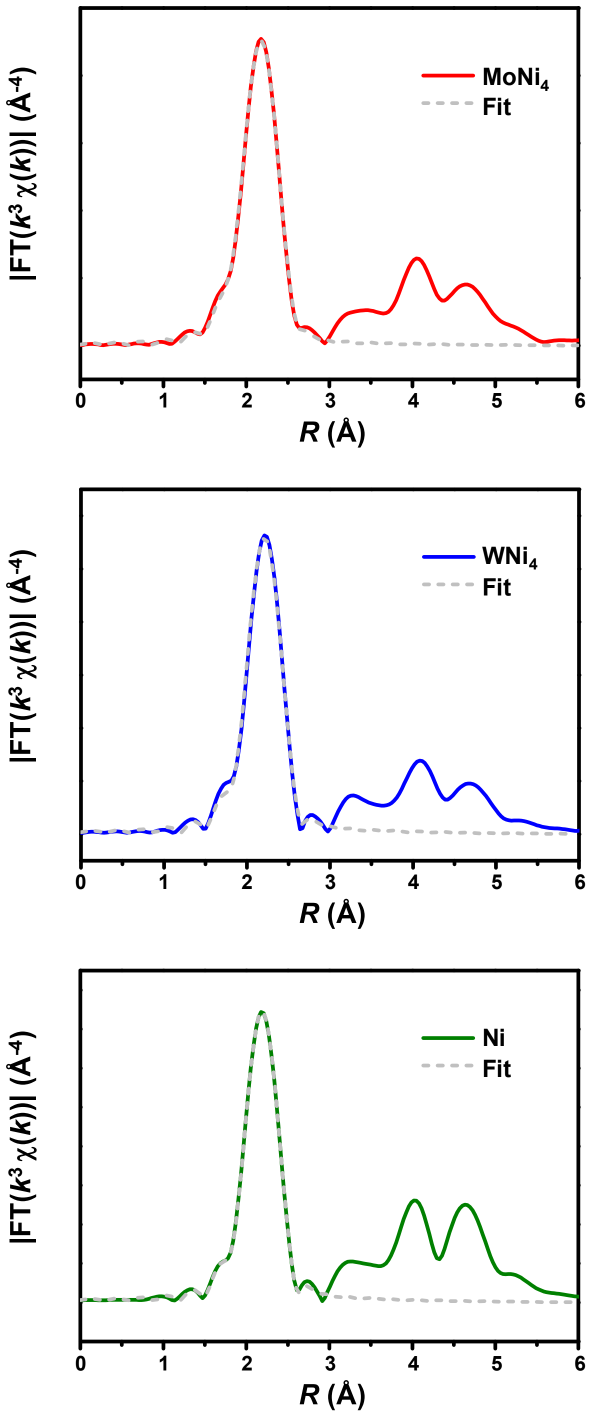

Supplementary Figure 9. The raw and fitting Fourier transform of k^3-weighted EXAFS spectra of Ni K-edge. a, $MoNi_4$. **b,** WNi_4. **c,** freshly-synthesized Ni. The first peak at about 2.2 Å in the EXAFS spectra can be attributed to the Ni-Ni or Ni-Mo(W) bonds[1, 2]. The fitting results (Supplementary Table 2) show that the coordination numbers in the first coordination shell of Ni atoms for $MoNi_4$, WNi_4 and freshly-synthesized Ni are 8.8, 8.6 and 10.5, respectively. We attribute the decreased coordination numbers of $MoNi_4$ and WNi_4 alloys to the alloying effect, smaller sizes and abundant steps compared with freshly-synthesized Ni.

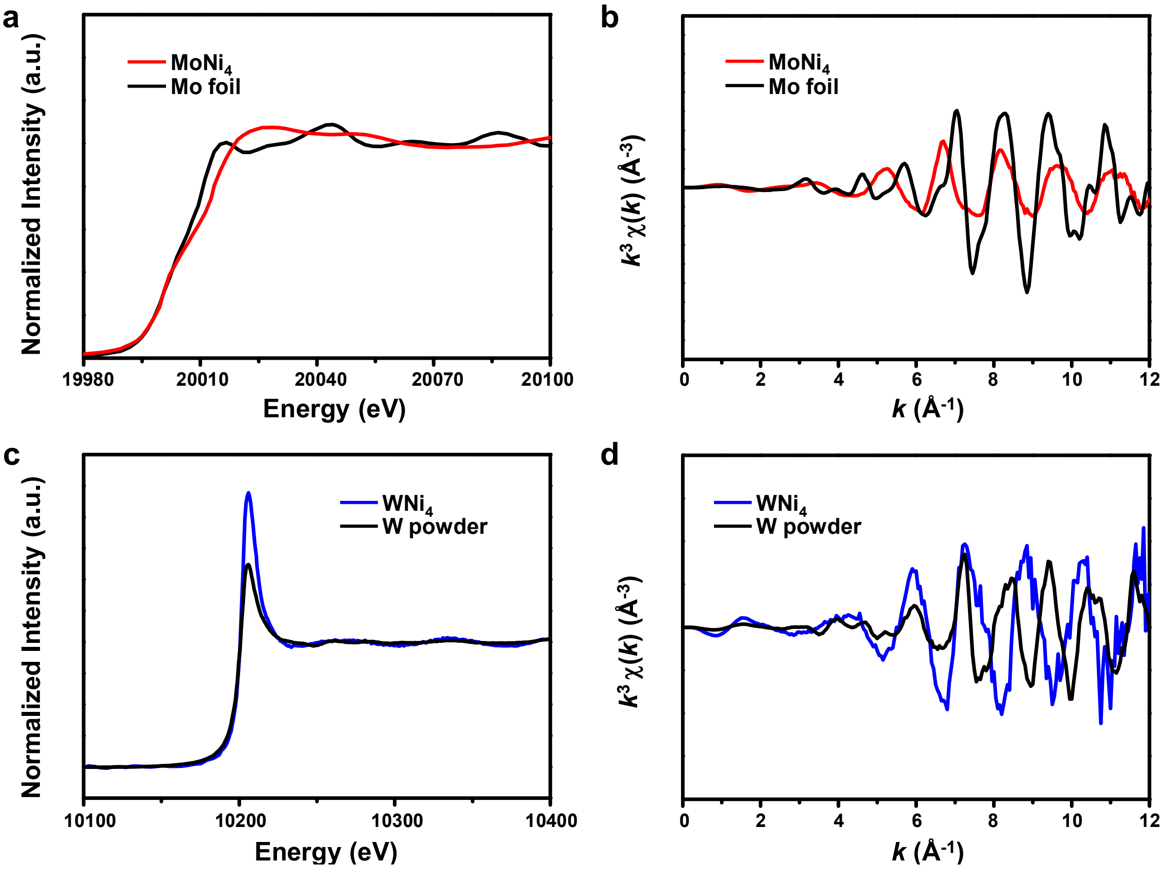

Supplementary Figure 10. XAS characterization. a, c, XANES spectra of Mo K-edge and W L_3-edge. **b, d,** EXAFS oscillation spectra of Mo K-edge and W L_3-edge. The XANES spectra and corresponding oscillation spectra exhibit the different electronic structure of Mo (W) atoms in alloys with that in bulk Mo foil (W powder), indicating the alloying structures are obtained.

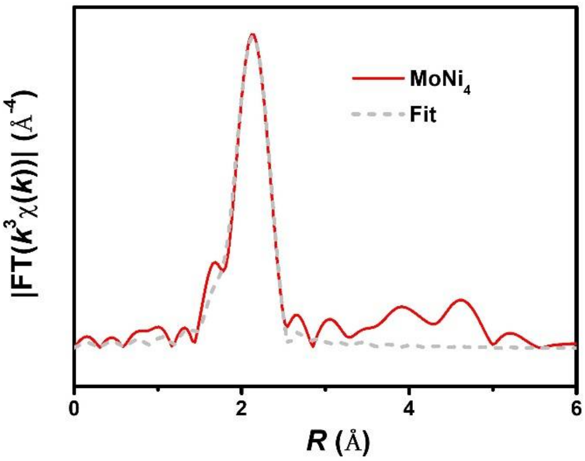

Supplementary Figure 11. The raw and fitting Fourier transform of k^3-weighted EXAFS spectra of Mo K-edge for $MoNi_4$.

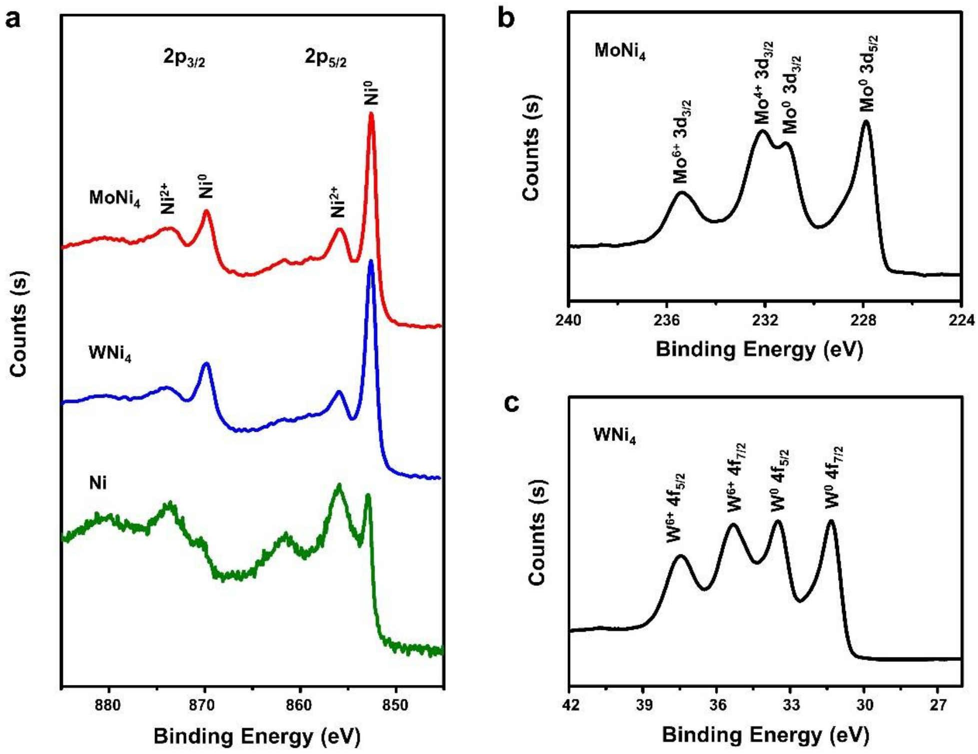

Supplementary Figure 12. XPS characterization. a, Ni 2*p* XPS of the studied catalysts. **b,** Mo 3*d* XPS of $MoNi_4$. **c,** W 4*f* XPS of WNi_4. For Ni 2*p* XPS spectra, the poignant peaks at 852.6 eV and 869.8 eV are assigned to Ni^0 and the weaker peaks located at 855.7 eV and 873.5 eV are indexed to Ni^{2+} (Ref. [3]). For Mo 3d XPS spectra, the peaks located at 227.9 eV, 231.1 eV, 232.1 eV and 235.3 eV are indexed to Mo^0 $3d_{5/2}$, Mo^0 $3d_{3/2}$, Mo^{4+} $3d_{3/2}$ and Mo^{6+} $3d_{3/2}$, respectively[3, 4]. For W 4f XPS spectra, the peaks located at 31.3 eV, 33.5 eV, 35.3 eV, and 37.5 eV are indexed to W^0 $4f_{7/2}$, W^0 $4f_{5/2}$, W^{6+} $4f_{7/2}$ and W^{6+} $4f_{5/2}$, respectively[5, 6]. Although with great care during XPS measurements, these samples are still inevitably exposed in air for a certain time, causing some surface oxidation of catalysts. Even so, our XPS results also reveal that single Ni metal possesses much higher Ni^{2+} peak than $MoNi_4$ and WNi_4 alloys, demonstrating that the surfaces of $MoNi_4$ and WNi_4 alloys are more difficult to be oxidized owing to the alloy effect, which would facilitate HOR catalysis.

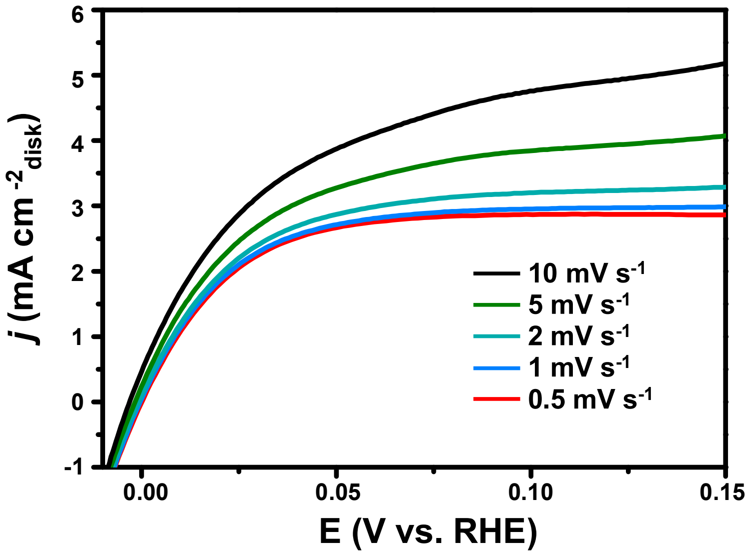

Supplementary Figure 13. The effect of sweep rates on HOR polarization curves. The figure displays the HOR polarization curves of $MoNi_4$ alloy at different sweep rates from 0.5 mV s^{-1} to 10 mV s^{-1} measured in H_2-saturated 0.1 M KOH. Rotation speed: 1600 rpm. It should be noted that large sweep rates cause increased current due to the contribution of capacitance current, which cannot reveal real HOR catalytic performance. Therefore, the sweep rates should be carefully noted for HOR measurement. Since the HOR polarization curves obtained at sweep rates of 1 mV s^{-1} and 0.5 mV s^{-1} show only slight change, the sweep of 0.5 mV s^{-1} is thought enough to reduce the capacitance contribution, which is adopted for all HOR performance measurements.

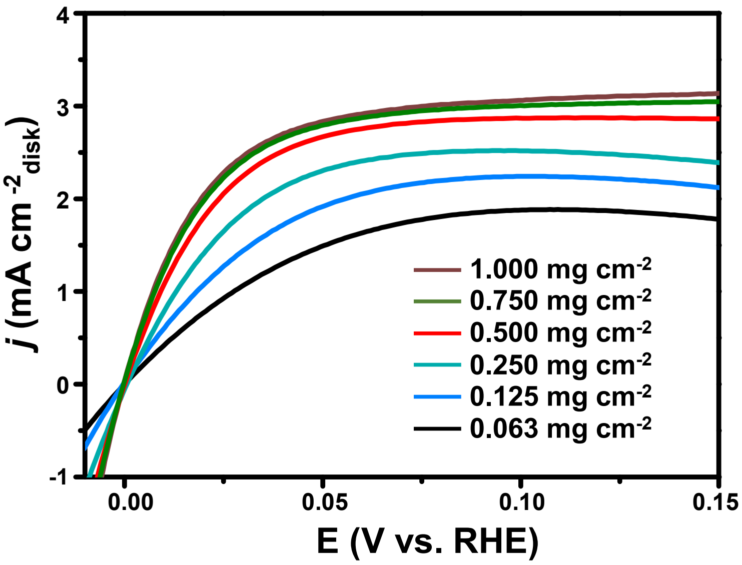

Supplementary Figure 14. The effect of catalyst loadings on HOR polarization curves. We test the HOR polarization curves of $MoNi_4$ with different loadings from 0.063 mg cm^{-2} to 1.000 mg cm^{-2} measured in H_2-saturated 0.1 M KOH. Sweep rate: 0.5 mV s^{-1}. Rotation speed: 1600 rpm. It can be seen that the polarization current increases as the catalyst loading increases until the loading achieves a value of 0.075 mg cm^{-2}. Here, we choose the 0.5 mg cm^{-2} loading for all HOR performance measurements.

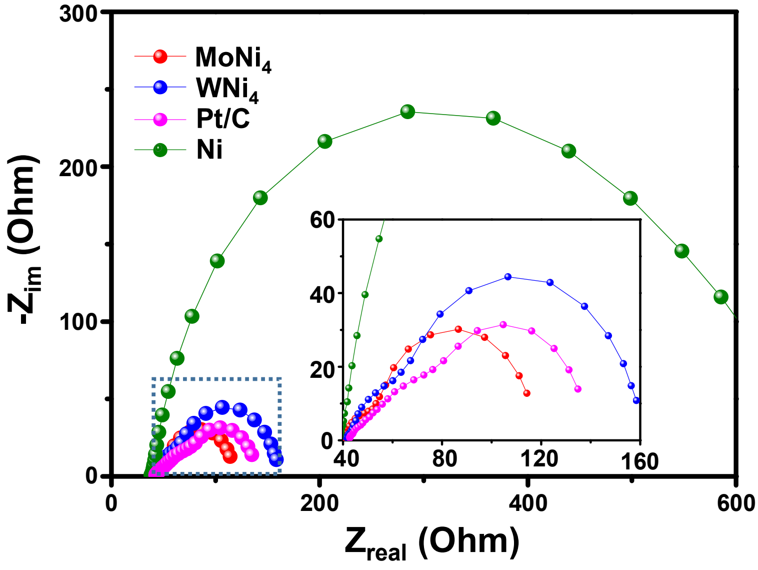

Supplementary Figure 15. EIS Nyquist plots of $MoNi_4$, WNi_4, freshly-synthesized Ni, and Pt/C. The EIS are conducted at 30 mV overpotential, which can offer the information of solution resistance (R_s) and charge transfer resistance (R_{ct}). The results demonstrate that the charge transfer resistance of $MoNi_4$ and WNi_4 alloys hugely decrease in contrast to freshly-synthesized Ni. Notably, the $MoNi_4$ alloy shows more rapid charge transfer than Pt/C, indicating its superior HOR activity.

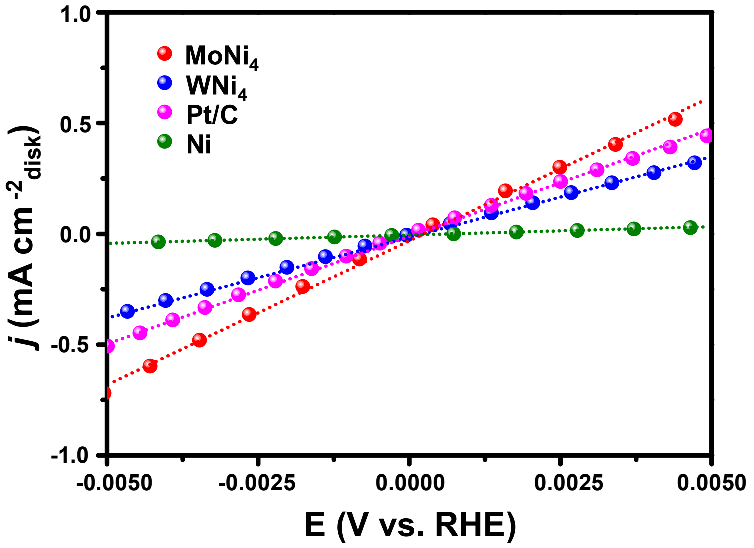

Supplementary Figure 16. Micropolarization regions (-5 mV to 5 mV) of $MoNi_4$, WNi_4, freshly-synthesized Ni, and Pt/C. In the Micropolarization region, Bulter-Volmer equation can be simplified to

$$j_0 = \frac{j}{\eta}\frac{RT}{F} \qquad \text{(Supplementary Equation 1)}$$

where j is the measured current density, η is the overpotential, R is the universal gas constant, T is the temperature, and F is Faraday's constant. Therefore, the exchange current density (j_0) can be obtained from the slope of the linear fitting of j-η curve in micropolarization regions. The obtained j_0 are consistent with the fitting results in Tafel regions (see Methods), which have been listed in Supplementary Table. 3.

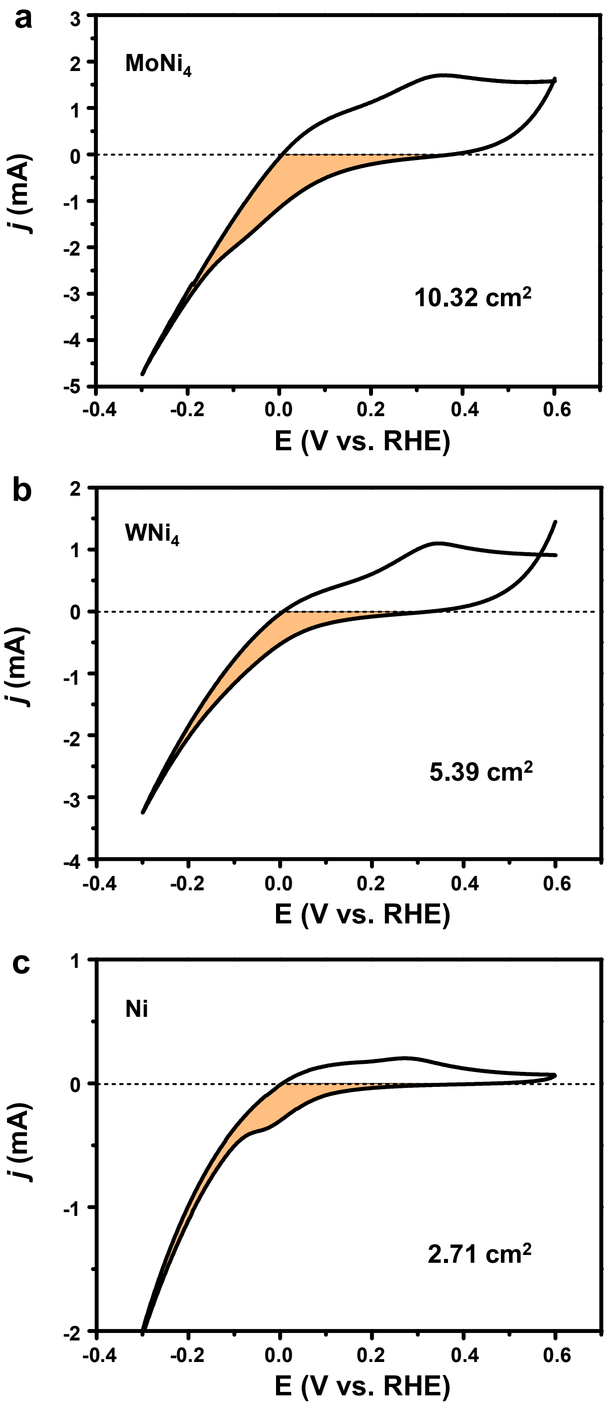

Supplementary Figure 17. Cyclic voltammetry (CV) curves measured in Ar-saturated 0.1 M KOH at a sweep rate of 50 mV s^{-1}. a, $MoNi_4$ alloy. **b**, WNi_4 alloy. **c**, freshly-synthesized Ni. Rotation speed: 1600 rpm. Electrochemical surface areas (ECSA) of the studied catalysts are estimated form the OH desorption region using a charge density of 514 μC cm^{-2}$_{Ni}$ for one monolayer of OH adsorption on Ni-based alloys[7].

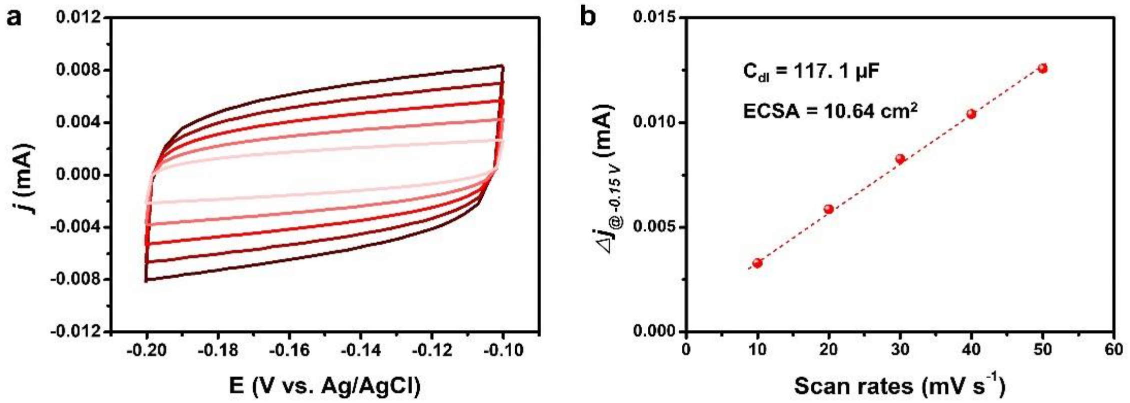

Supplementary Figure 18. ECSA by non-faradaic double layer capacitance obtained in CH_3CN with 0.15 M KPF_6. a, CV curves of $MoNi_4$ alloy collected at various scan rates ranging from 10 to 50 mV s^{-1}. **b**, The corresponding linear fitting of scan rates versus difference between the anodic and cathodic current at -0.15 V vs. Ag/AgCl. We measure the ECSA of $MoNi_4$ by the double layer capacitance method on the basis of cyclic voltammetry in a nonaqueous aprotic KPF_6–CH_3CN electrolyte[8]. The electrochemical double layer capacitance (C_{dl}) is extracted from **b**. The ECSA can be calculated according to the following equation: ECSA = C_{dl}/C_s, where C_s is the specific capacitance of a flat smooth surface of the electrode material, which was determined to be 11 μF cm^{-2}. The result gives an ECSA value of 10.64 cm^2 for the $MoNi_4$ catalyst loaded on the glassy carbon electrode (0.196 cm^2), consistent with the above result of 10.32 cm^2 quantified by the redox reaction of surface metals.

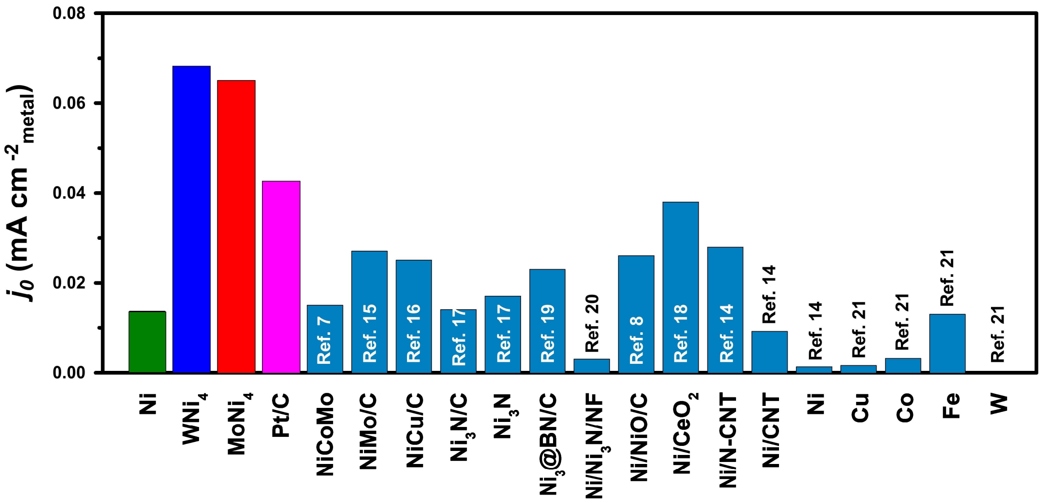

Supplementary Figure 19. Comparison of the intrinsic HOR activities—ECSA normalized exchange current density—of $MoNi_4$, WNi_4, freshly-synthesized Ni, commercial Pt/C and most reported PGM-free HOR catalysts measured in alkaline electrolyte (pH 13). The intrinsic HOR activities of $MoNi_4$ and WNi_4 catalysts are 0.065 and 0.068 mA cm^{-2}, respectively, which is best PGM-free HOR catalysts measured in alkaline electrolytes, including various Ni-based compounds listed in the Supplementary Table 4.

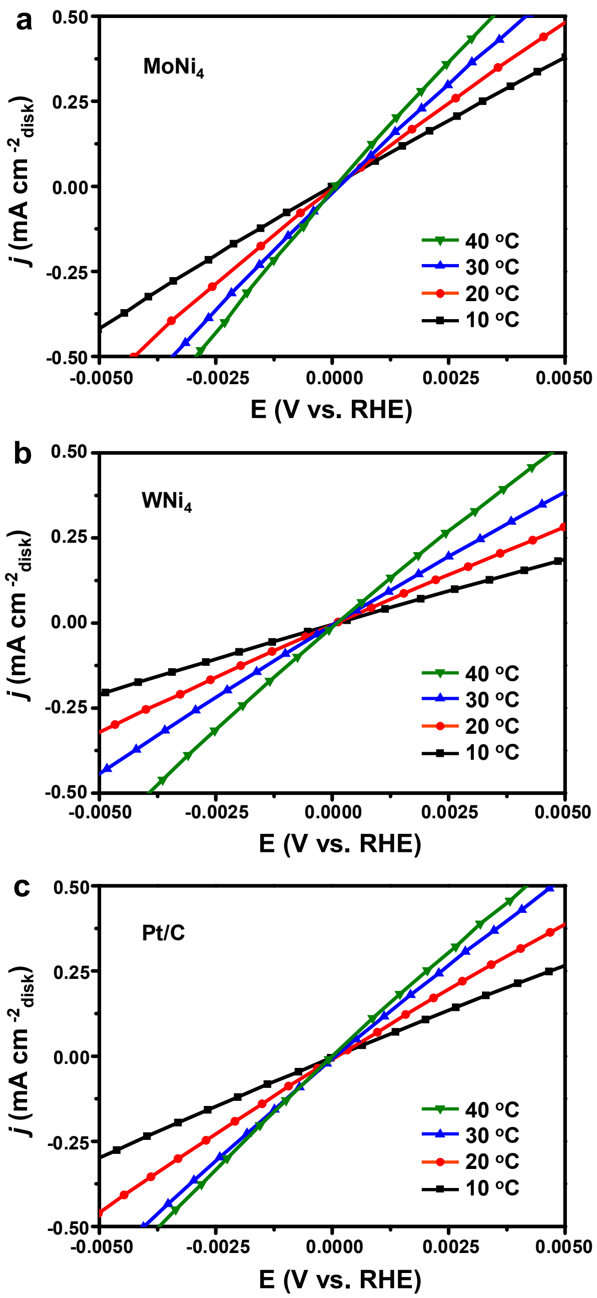

Supplementary Figure 20. Micropolarization region (-5 mV to 5 mV) of the studied catalysts measured at different temperatures from 10 °C to 40 °C. a, $MoNi_4$ alloy. **b**, WNi_4 alloy. **c**, Pt/C. Sweep rate: 0.5 mV s^{-1}. Rotation speed: 1600 rpm. The electrochemical activation energy (E_a) of HOR/HER can be calculated by the Arrhenius equation:

$$\log j_0 = \frac{-Ea}{\ln 10 \times R \times T} + \text{const} \qquad \text{(Supplementary Equation 2)}$$

where j_0 is the exchange current density at different temperatures (T), and R is the universal gas constant. Therefore, E_a can be calculated from the slope of Arrhenius plot (Figure 3f).

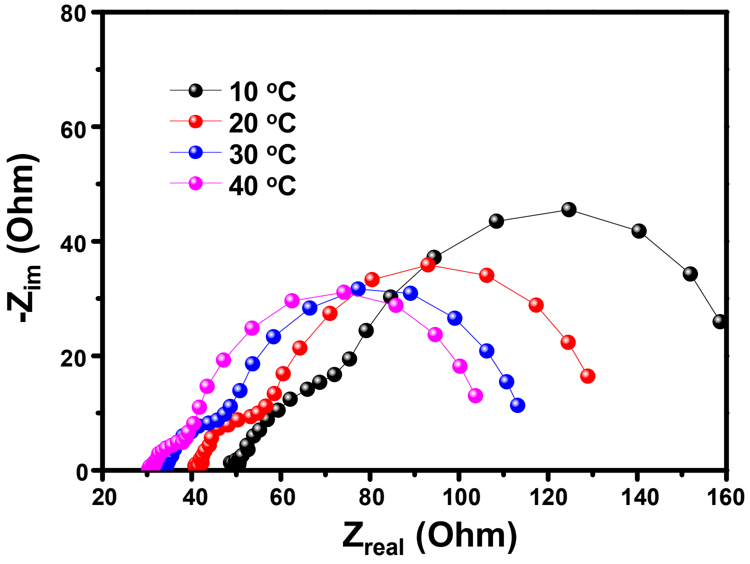

Supplementary Figure 21. EIS Nyquist plots of $MoNi_4$ at different temperatures. It can be seen that the solution resistances increase as test temperatures become lower, which are collected for *i*R correction in Supplementary Fig. 17.

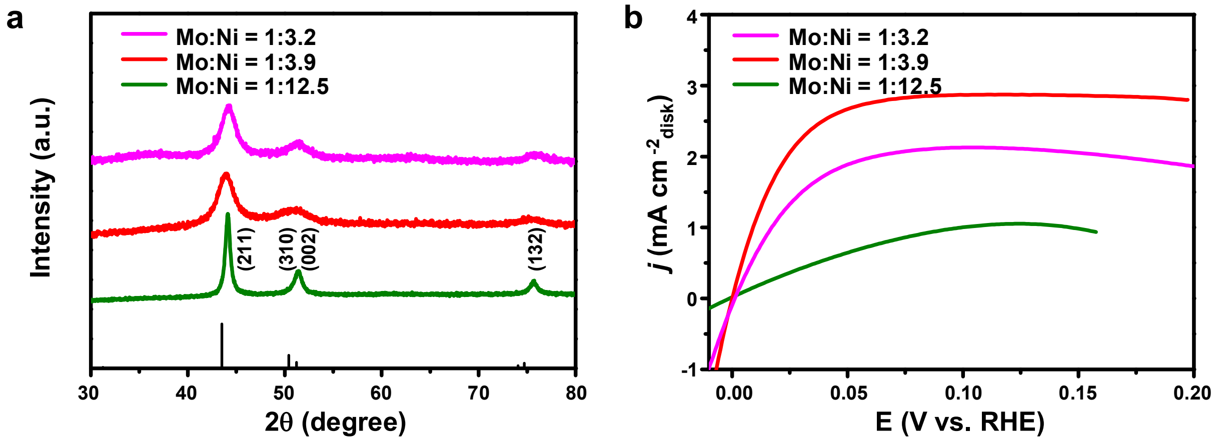

Supplementary Figure 22. Effect of different Mo:Ni ratios on HOR activities. a, XRD patterns. The inserted lines in **a** are indexed to tetragonal $MoNi_4$ (*JCPDS* 65-5480). **b,** HOR polarization curves. Sweep rate: 0.5 mV s^{-1}. Rotation speed: 1600 rpm. The Mo-Ni alloys with different Mo:Ni ratios were synthesized by change the addition amounts of $(NH_4)_6Mo_7O_{24}\cdot4H_2O$ during the synthesis of Mo-doped $Ni(OH)_2$ precursors, in which the Mo:Ni ratios were determined by the ICP-AES results. The catalyst with the Mo:Ni ratio of about 1:4 show best HOR activity, indicating the optimized alloying structure of tetragonal $MoNi_4$.

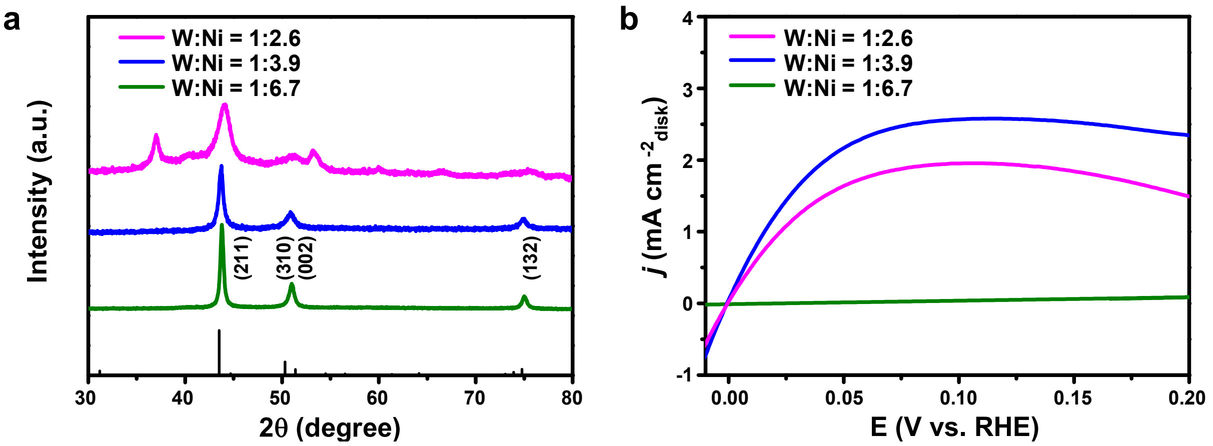

Supplementary Figure 23. Effect of different W:Ni ratios on HOR activities. a, XRD patterns. The inserted lines in **a** are indexed to tetragonal WNi_4 (*JCPDS* 65-2673). **b,** HOR polarization curves. Sweep rate: 0.5 mV s^{-1}. Rotation speed: 1600 rpm. The W-Ni alloys with different W:Ni ratios were synthesized by change the addition amounts of $(NH_4)_{10}W_{12}O_{41}\cdot xH_2O$ during the synthesis of W-doped $Ni(OH)_2$ precursors, in which the W:Ni ratios were determined by the ICP-AES results. The catalyst with the W:Ni ratio of about 1:4 show best HOR activity, indicating the optimized alloying structure of tetragonal WNi_4.

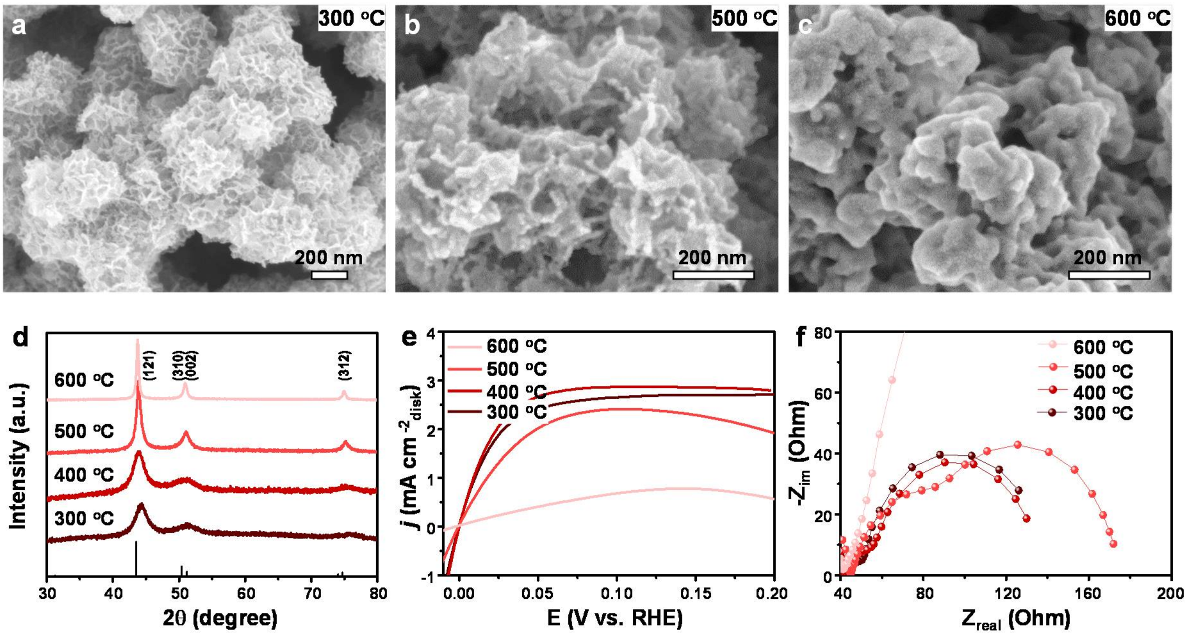

Supplementary Figure 24. Physical characterization and HOR activities of Mo-Ni alloys reduced at different temperatures. a-c, SEM images of catalysts obtained at 300 °C (**a**), 500 °C (**b**) and 600 °C (**c**). **d,** XRD patterns. The inserted lines are indexed to $MoNi_4$ (*JCPDS* 65-5480). **e**, HOR polarization curves. **f**, EIS Nyquist plots. The Mo-doped $Ni(OH)_2$ precursors are annealed at different temperatures from 300 °C to 600 °C. As shown in the **a-c**, increased aggregations are detected as the temperatures increase. The catalyst annealed at 400 °C shows best HOR performance (**e**) and lowest charge transfer resistance (**f**).

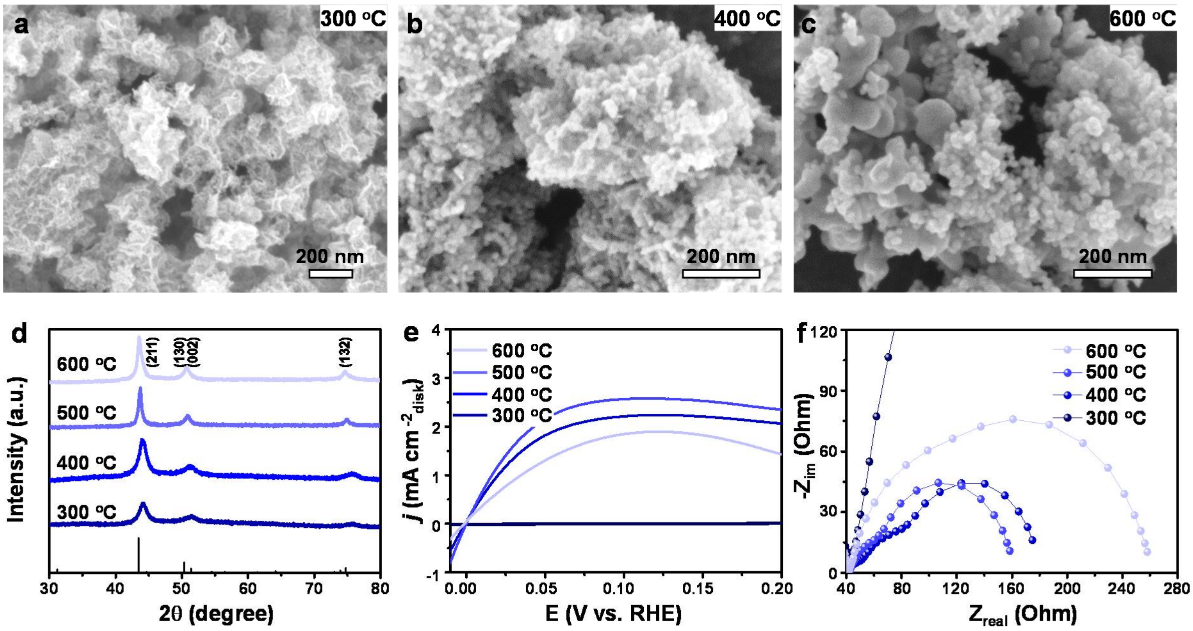

Supplementary Figure 25. Physical characterization and HOR activities of W-Ni alloys reduced at different temperatures. a-c, SEM images of catalysts obtained at 300 °C (**a**), 400 °C (**b**) and 600 °C (**c**). **d,** XRD patterns. The inserted lines are indexed to WNi_4 (*JCPDS* 65-2673). **e**, HOR polarization curves. **f**, EIS Nyquist plots. The W-doped $Ni(OH)_2$ precursors are annealed at different temperatures from 300 °C to 600 °C. Based on the morphology characterization in **a-c** and XRD patterns in **d**, catalysts annealed at 300 °C and 400 °C show no complete decomposition from W-doped $Ni(OH)_2$ precursors to form WNi_4 alloy. Yet, higher temperature (600 °C) causes a severe aggregation of the catalysts, leading to activity decay. Therefore, the alloy annealed at 500 °C possesses best HOR activities (**e**) and lowest charge transfer resistance (**f**).

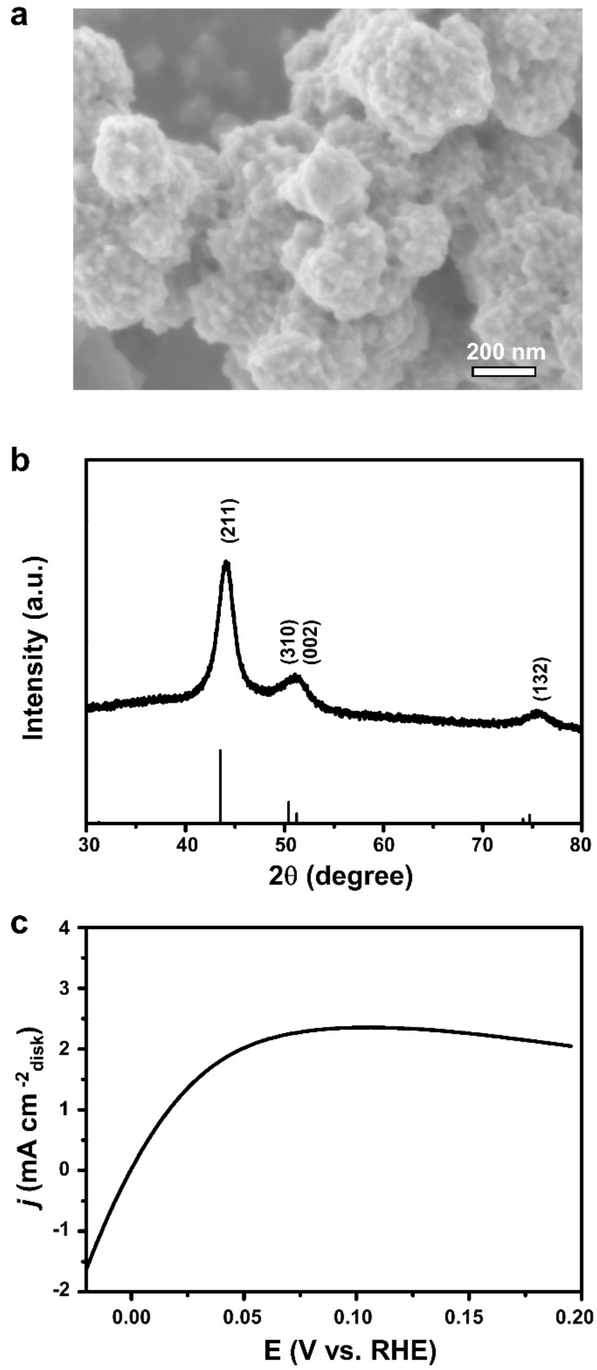

Supplementary Figure 26. Physical characterization and HOR activities of $MoNi_4$ nanoparticles. **a**, SEM image. **b**, XRD pattern. The inserted lines are indexed to $MoNi_4$ (*JCPDS* 65-5480). **c**, HOR polarization curve. The $MoNi_4$ nanoparticles was synthesized on the basis of a previous method[9]. The Mo:Ni ratio is determined to 1:3.98 by ICP-AES test.

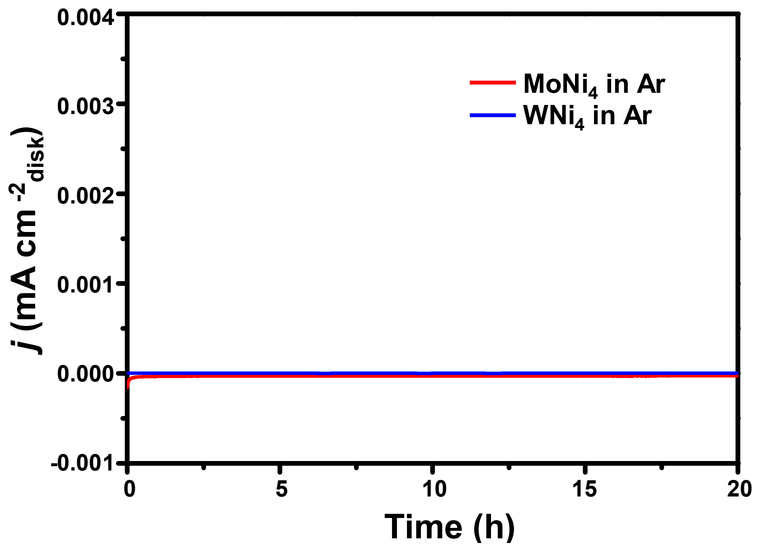

Supplementary Figure 27. Chronoamperometry (*j* ~ t) responses recorded on $MoNi_4$ and WNi_4 alloys at a 60 mV overpotential in Ar-saturated 0.1 M KOH. The curves measured in Ar atmosphere show only negligible current density for $MoNi_4$ and WNi_4 alloys, confirming the oxidation current in Figure 4c are assigned to the hydrogen oxidation.

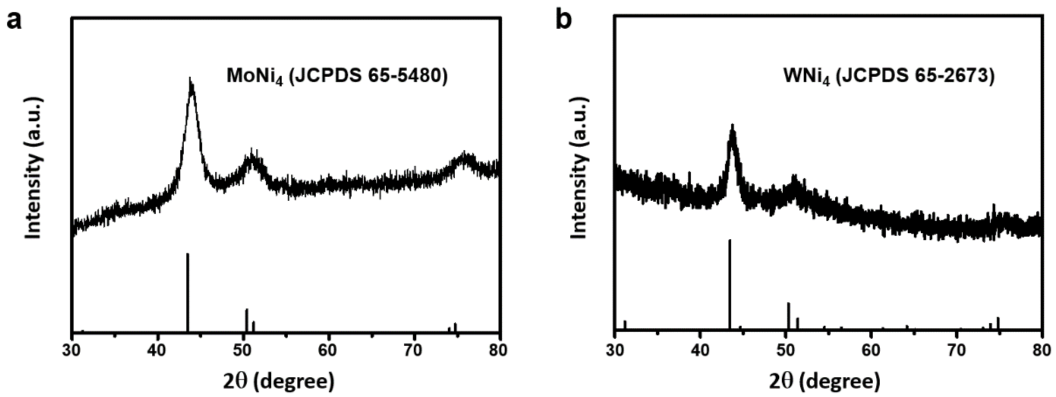

Supplementary Figure 28. XRD patterns. a, $MoNi_4$ after HOR test. The inserted lines are indexed to $MoNi_4$ (*JCPDS* 65-5480). **b**, WNi_4 after HOR test. The inserted lines are indexed to WNi_4 (*JCPDS* 65-2673). As shown above, the crystal phases of $MoNi_4$ and WNi_4 alloys are well maintained after HOR test.

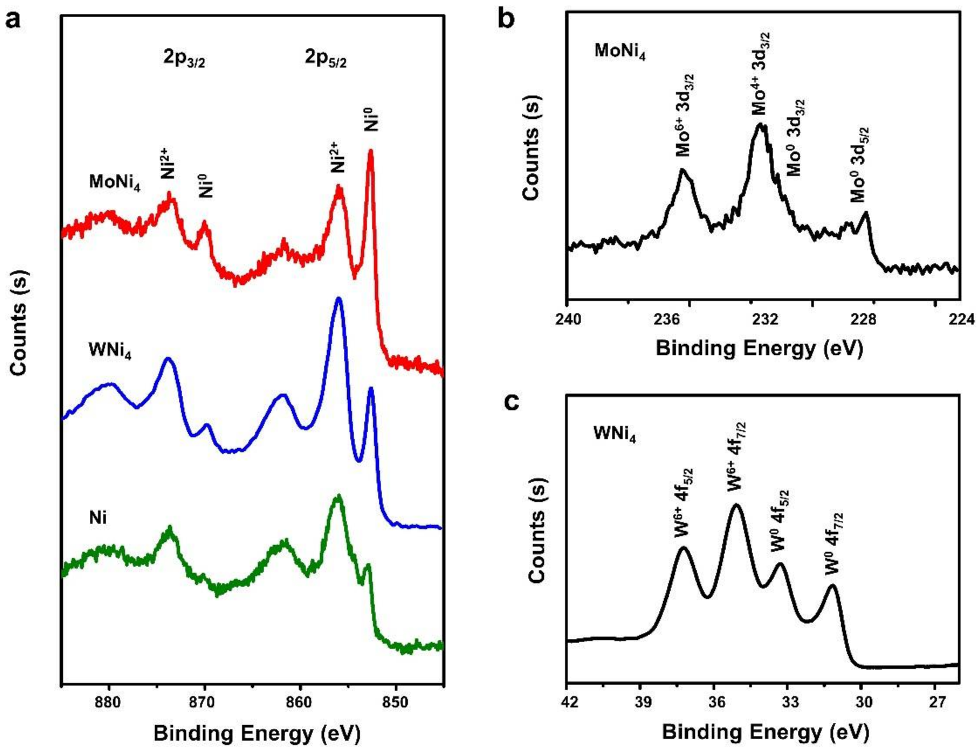

Supplementary Figure 29. XPS characterization after HOR test. **a**, Ni 2p XPS of $MoNi_4$, WNi_4 and Ni after HOR test. **b**, Mo 3d XPS of $MoNi_4$ after HOR test. **c**, W 4f XPS of WNi_4 after HOR test. We find that the $MoNi_4$ and WNi_4 alloys still have strong Ni^0 signals even after stability test for 3 h. The Ni^{2+} signals come from their surface oxidation when exposing in air and in alkali. By contrast, Ni^0 signals almost disappeared for single Ni catalyst, with only Ni^{2+} signals left. Moreover, the Mo 3d and W 4f XPS spectra both only show slight change, suggesting the marked robustness of the $MoNi_4$ and WNi_4 alloyed catalysts.

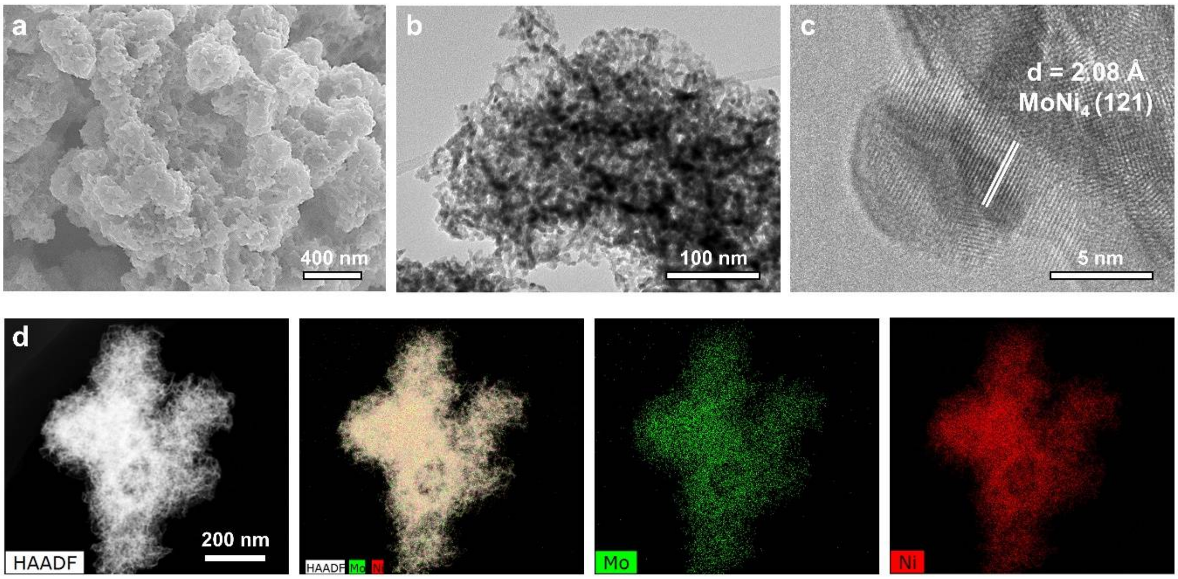

Supplementary Figure 30. Physical characterization of $MoNi_4$ after stability test. a, SEM image. **b**, TEM image. **c**, HRTEM image. **d**, STEM-EDX elemental mappings. As shown above, the morphology and structure of $MoNi_4$ alloy are well maintained after HOR test.

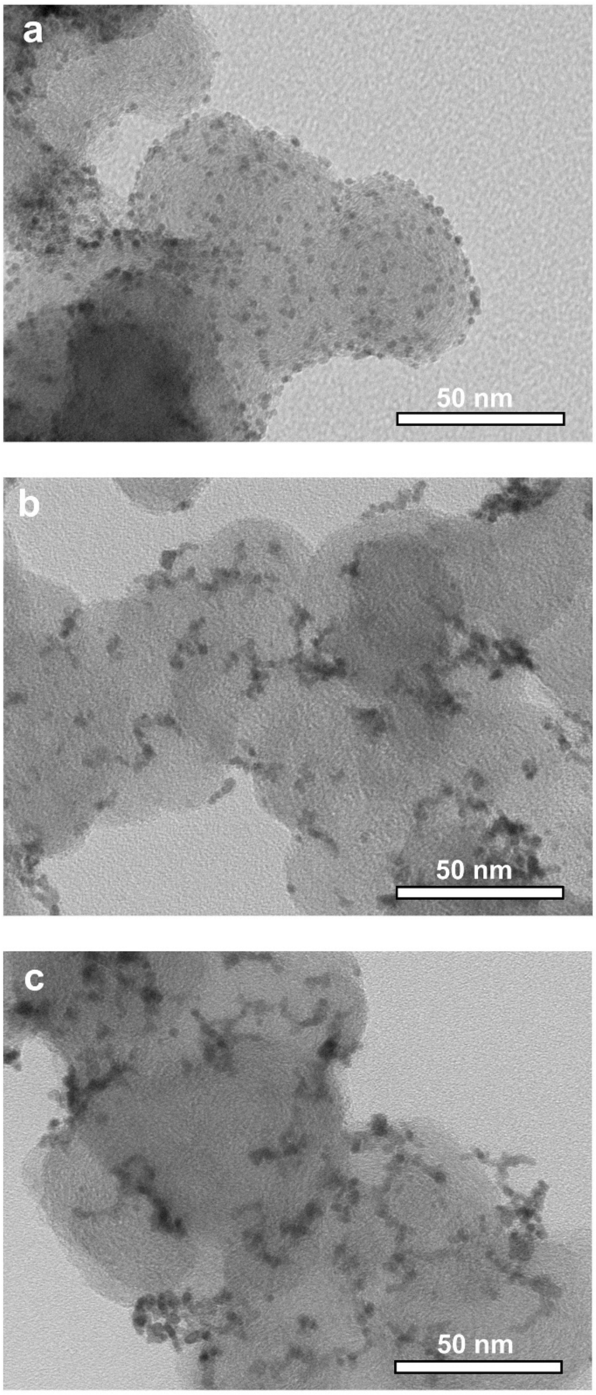

Supplementary Figure 31. TEM images for commerical Pt/C after different time of stability test. **a**, 0 h. **b**, 10 h. **c**, 20 h. A clear particle size agglomeration can be found as reaction time prolongs. After reacting from 0 h to 20 h, the Pt particle size grows obviously and forms agglomerated clusters, which thus results in the reduction of active surface areas and further activity degradation, in agreement with previous observations on Pt/C catalyst [10, 11].

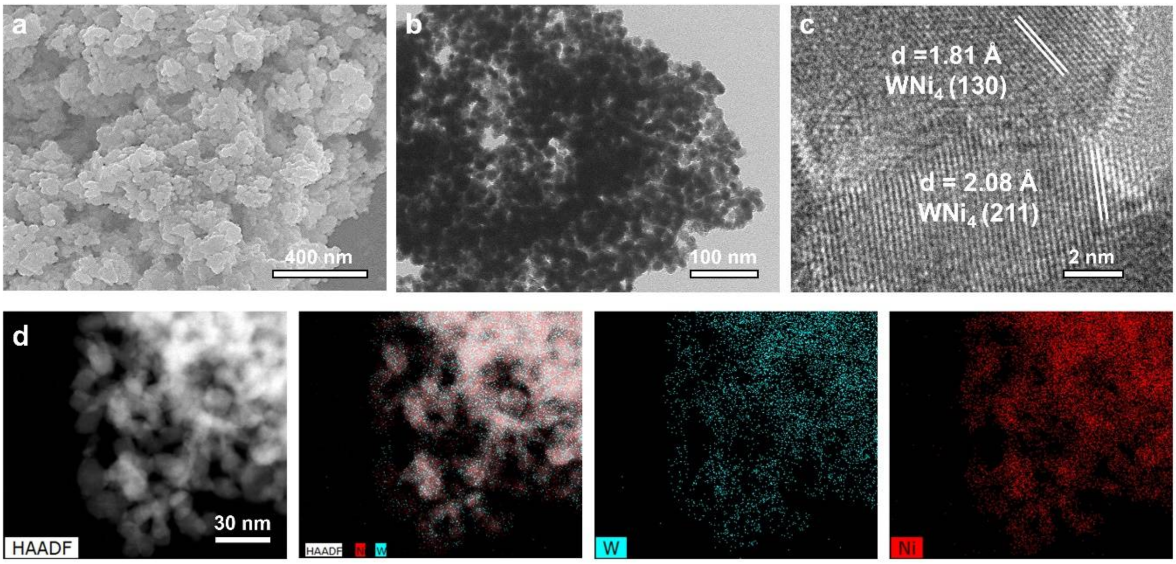

Supplementary Figure 32. Physical characterization of WNi_4 after stability test. a, SEM image. **b**, TEM image. **c**, HRTEM image. **d**, STEM-EDX elemental mappings. Based on the characterization above, the morphology and structure of WNi_4 alloy are well maintained after HOR test.

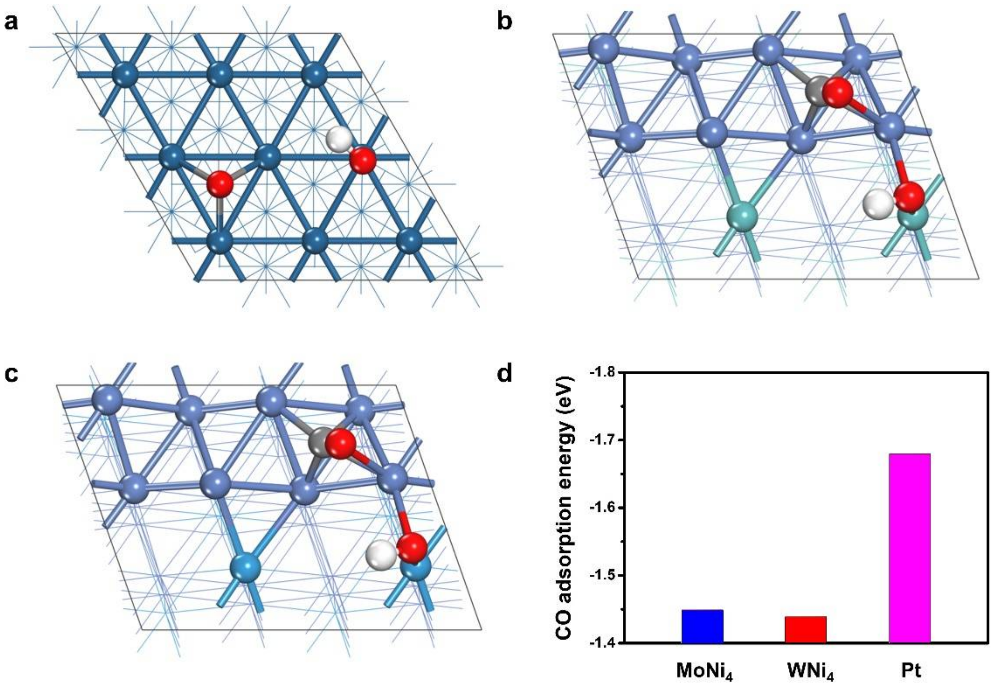

Supplementary Figure 33. Model and calculated results of CO adsorption on catalysts. a, Pt(111). **b**, $MoNi_4$(211). **c**, WNi_4(211). **d**, Calculated CO adsorption energies on catalysts. In the alkaline condition, the CO molecule could be adsorbed on the catalyst surface as following steps[12]:

$$* + OH^- \rightarrow OH^*$$

$$OH^* + CO \rightarrow (OH+CO)^*$$

The adsorption energies of CO molecule were calculated according to $E_{CO\text{-}ads}=E_{CO@cat.}-E_{cat.}-E_{CO}$, where $E_{CO\text{-}ads}$ represents the energy of catalysts with the adsorbed CO, while $E_{cat.}$, E_{CO} stand for the energies of the catalysts and CO molecule. A more negative adsorption energy indicates a larger binding energy.

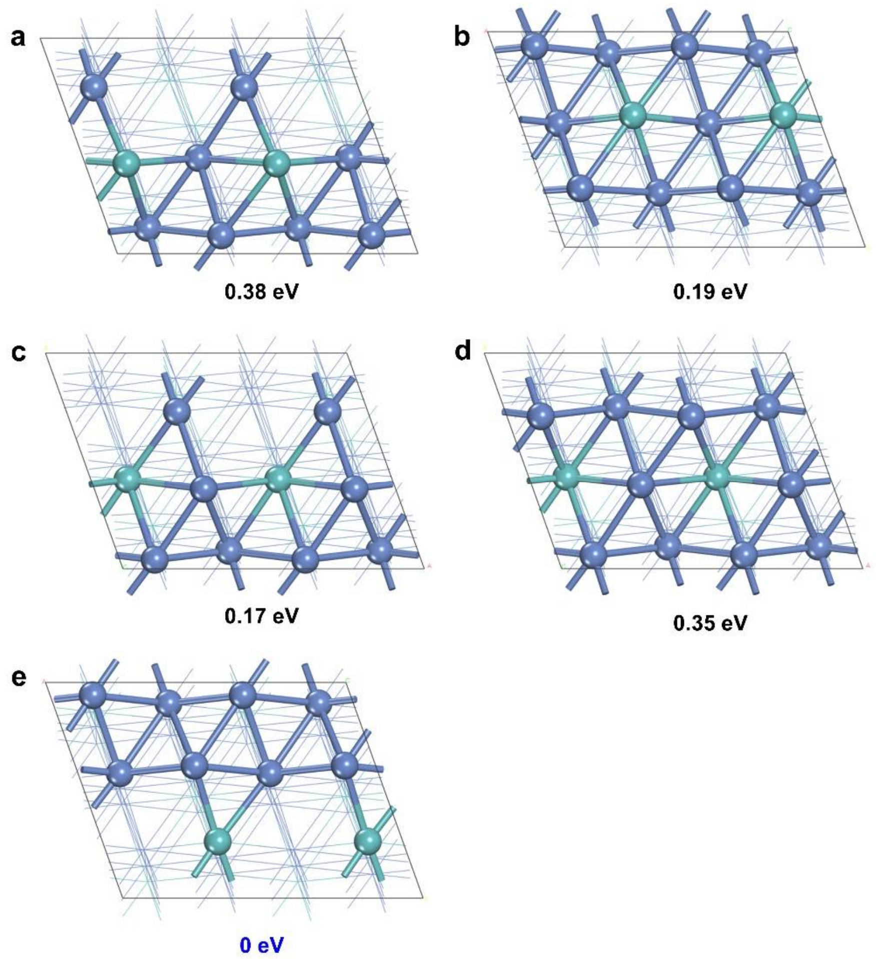

Supplementary Figure 34. Computed structures of the $MoNi_4$(211) planes with different exposed atomic configurations. Bottom values indicate the energy differences relative to the most stable structure. The gray and green spheres represent Ni and Mo atoms, respectively.

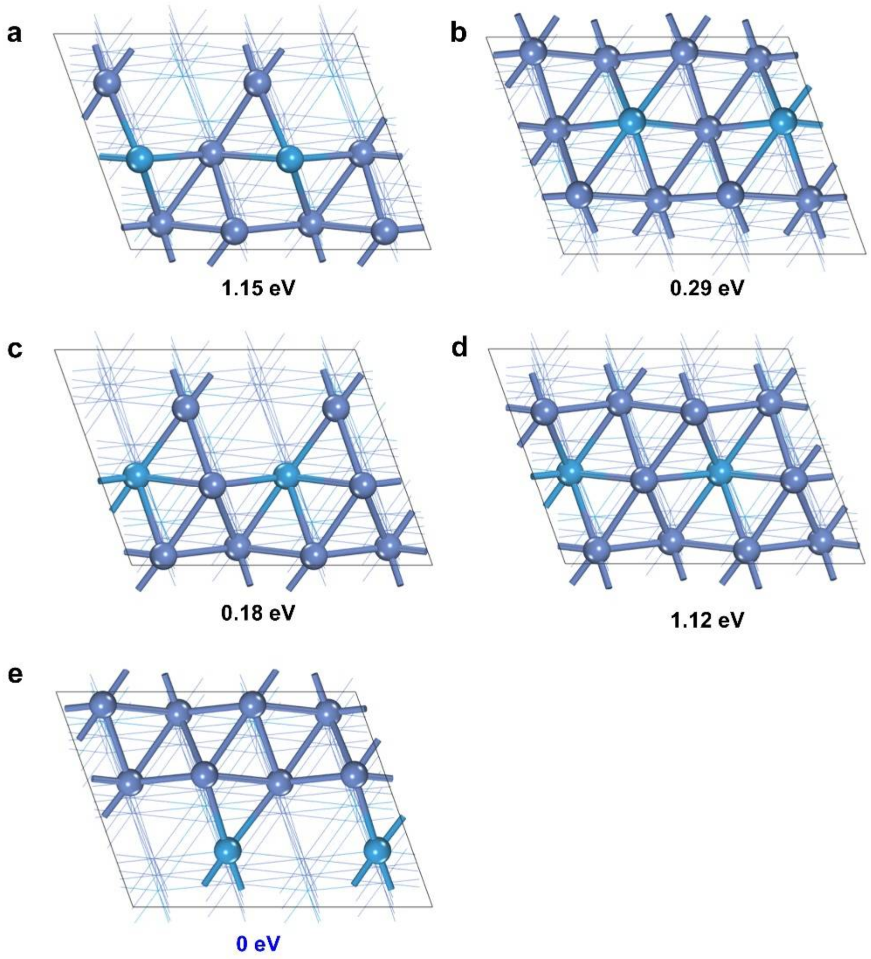

Supplementary Figure 35. Computed structures of the WNi_4(211) planes with different exposed atomic configurations. Bottom values indicate the energy differences relative to the most stable structure. The gray and blue spheres represent Ni and W atoms, respectively.

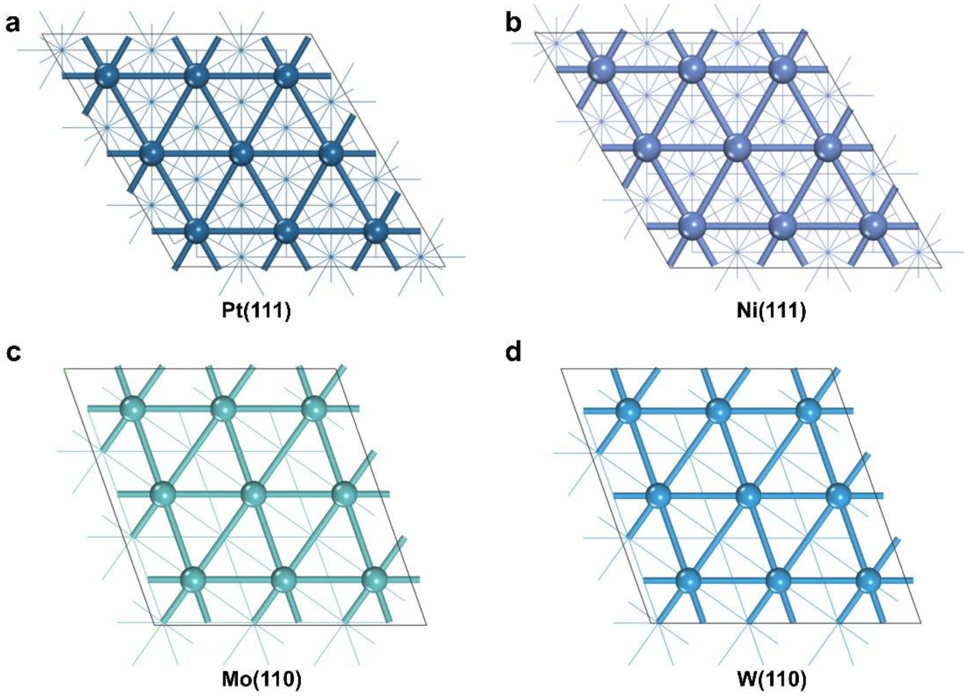

Supplementary Figure 36. Model of single metal. **a**, Pt(111). **b**, Ni(111). **c**, Mo(110). **d**, W(110). The cyan, gray, green and blue spheres represent Pt, Ni, Mo and W atoms, respectively.

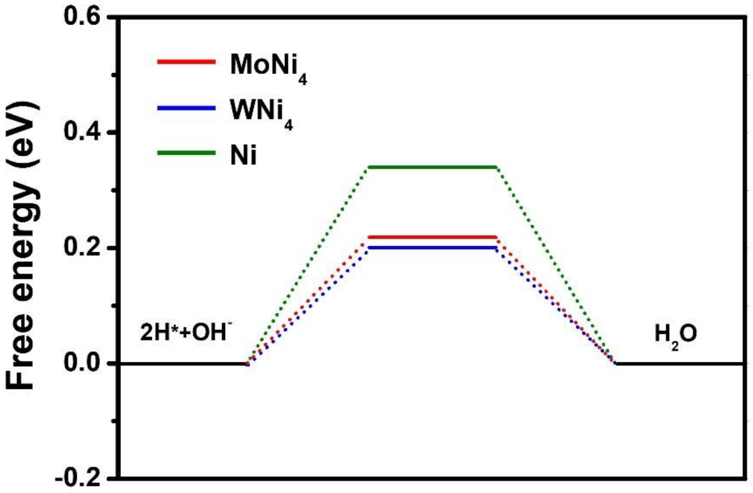

Supplementary Figure 37. Calculated free energy diagram for the Volmer step.

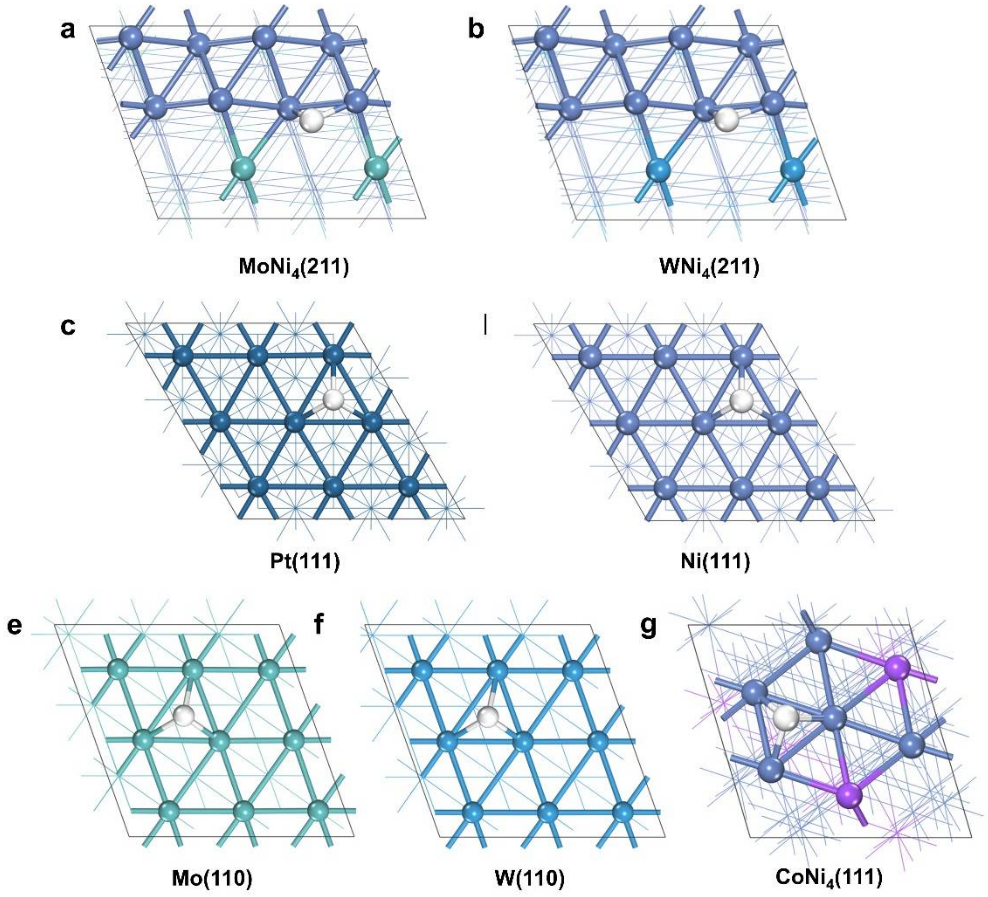

Supplementary Figure 38. Model of H adsorption on catalysts. a, $MoNi_4(211)$. **b**, $WNi_4(211)$. **c**, Pt(111). **d**, Ni(111). **e**, Mo(110). **f**, W(110). **g**, $CoNi_4(111)$. The gray, green, blue, cyan, purple and white spheres represent Ni, Mo, W, Pt, Co and H atoms, respectively.

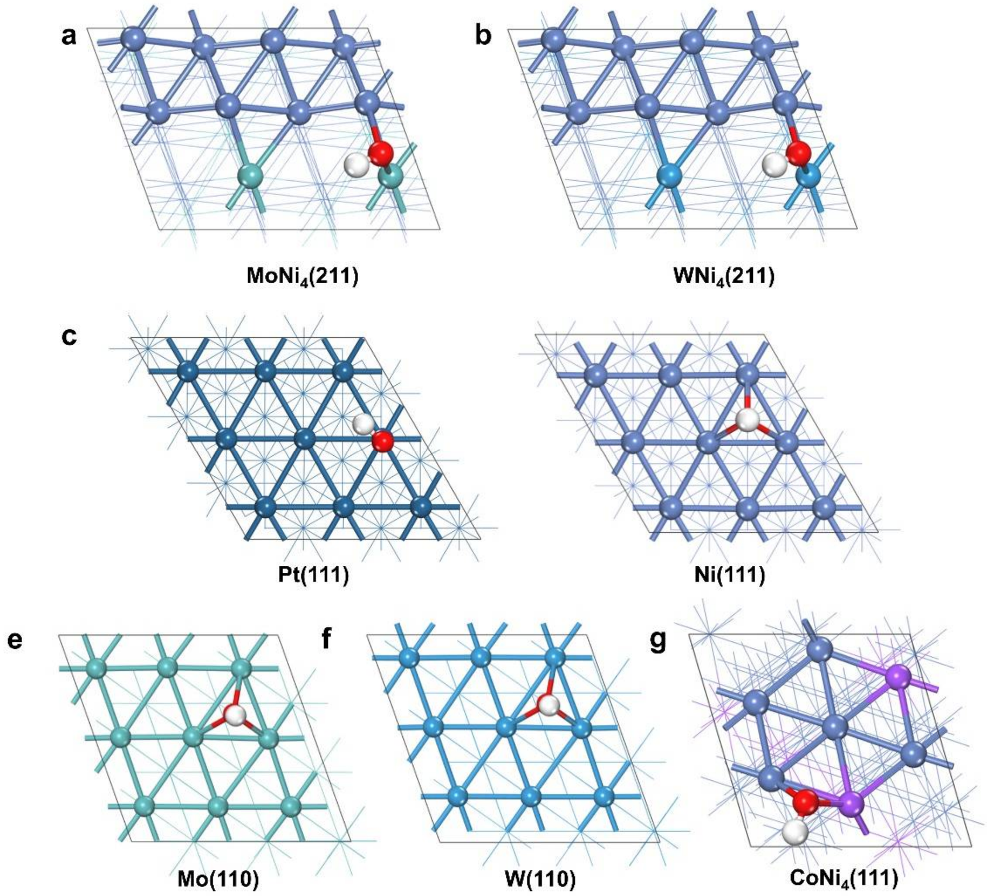

Supplementary Figure 39. Model of OH adsorption on catalysts. a, $MoNi_4$(211). **b**, WNi_4(211). **c**, Pt(111). **d**, Ni(111). **e**, Mo(110). **f**, W(110). **g**, $CoNi_4$(111). The gray, green, blue, cyan, purple, red and white spheres represent Ni, Mo, W, Pt, Co, O and H atoms, respectively.

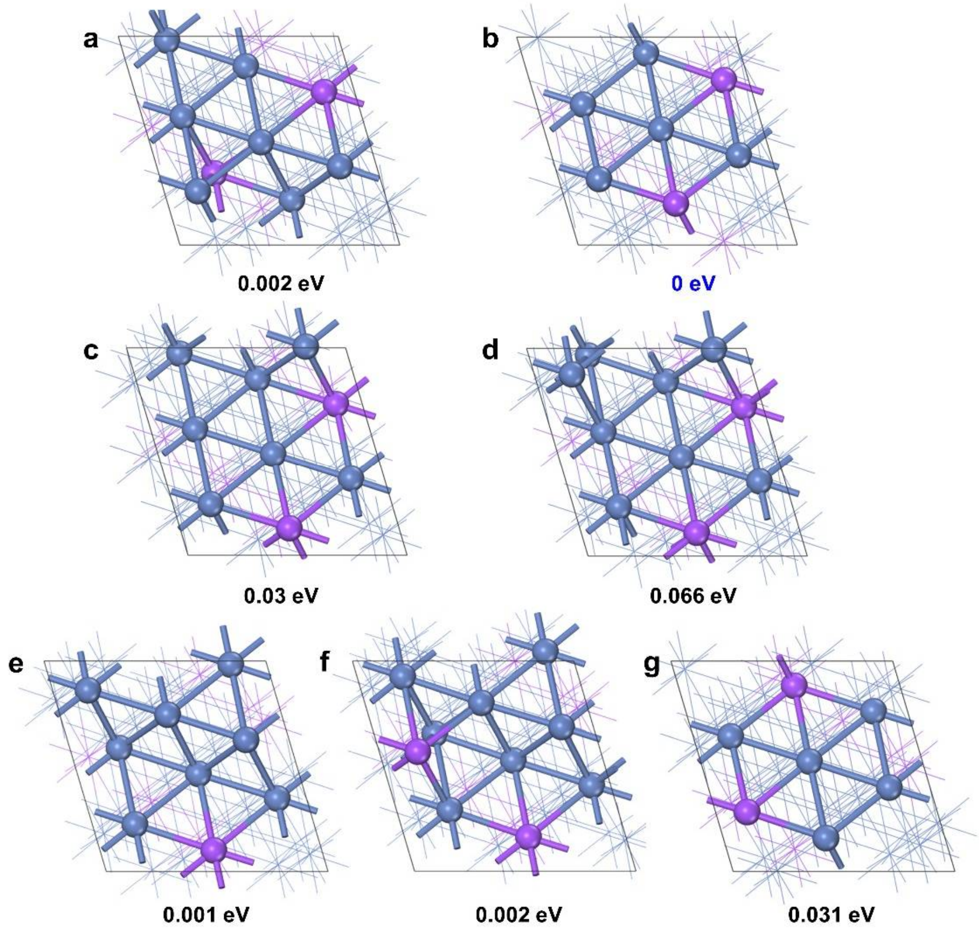

Supplementary Figure 40. Computed structures of the $CoNi_4(111)$ planes with different exposed atomic configurations. Bottom values indicate the energy differences relative to the most stable structure. The gray and purple spheres represent Ni and Co atoms, respectively. It is noted that the structures of $FeNi_4(111)$ are similar with those of $CoNi_4(111)$.

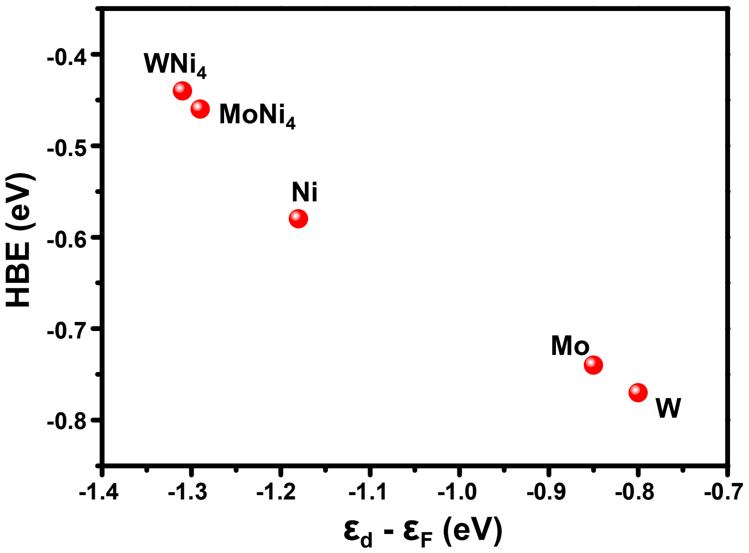

Supplementary Figure 41. Calculated shifts in the *d*-band center (ε_d) with respect to the Fermi level (ε_F) and hydrogen binding energy (HBE) on $MoNi_4$(211),WNi_4(211), Ni(111), Mo(110) and W(110). The *d*-band theory has been well adopted in metal–H adsorption system[13], where the *d*-band center describes the relative filling of the hybridized *(d–s)* and antibonding *(d–s)** states of the metal–H band. The negative shift of *d*-band center relative to Fermi level results antibonding part of the band is pulled down below the Fermi level, further leading to weaker binding between the metal atoms and the adsorbed hydrogen atoms. The calculated $MoNi_4$ and WNi_4 alloys show noteworthy negative shift of *d*-band center relative to Fermi level, which is consistent with the experimental observation in UPS (Figure 5a). It can be seen that Mo(W)-doping in alloys can weaken the too strong hydrogen binding energy of initial Ni metal, resulting in a great improvement of HOR activities.

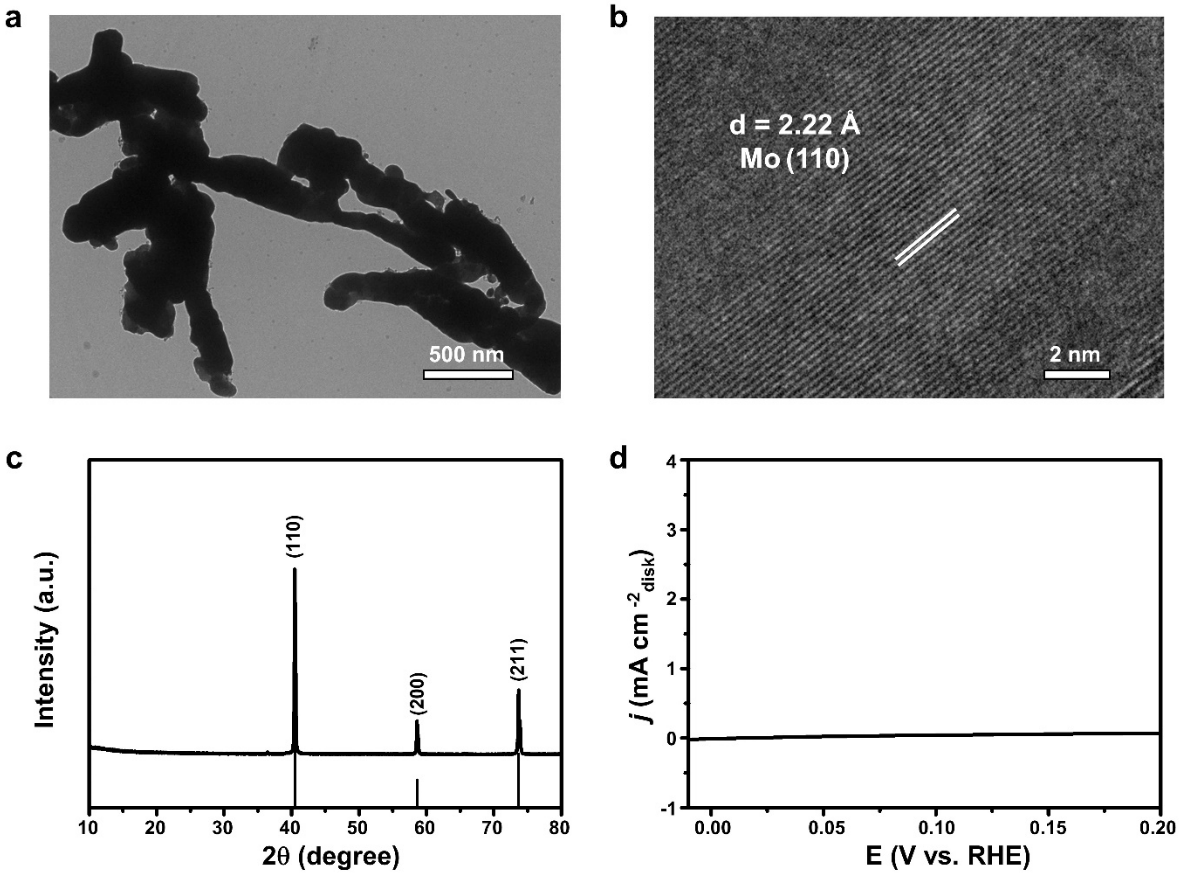

Supplementary Figure 42. Physical characterization and HOR activity of Mo metal. a, SEM image. **b**, HRTEM image. **c**, XRD pattern. **d**, HOR polarization curve. The inserted lines in **c** are indexed to Mo (*JCPDS* 42-1120). The HRTEM and XRD suggest that the nano-sized Mo metal has been well synthesized. However, no HOR current can be detected on the obtained Mo metal.

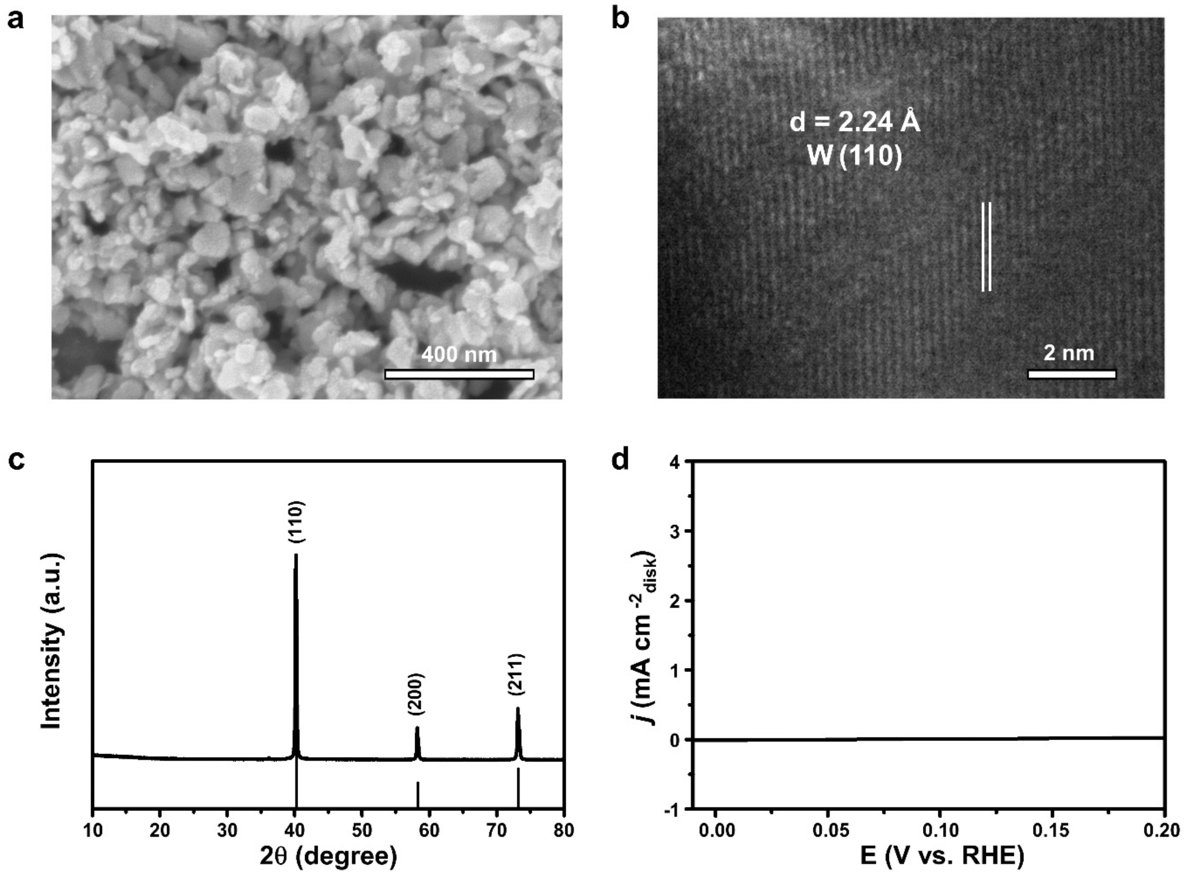

Supplementary Figure 43. Physical characterization and HOR activity of W metal. a, SEM image. **b**, HRTEM image. **c**, XRD pattern. **d**, HOR polarization curve. The inserted lines in **c** are indexed to W (*JCPDS* 04-0806). The HRTEM and XRD confirm the synthesis of nano-sized W metal. However, the obtained W metal show no HOR activity**.**

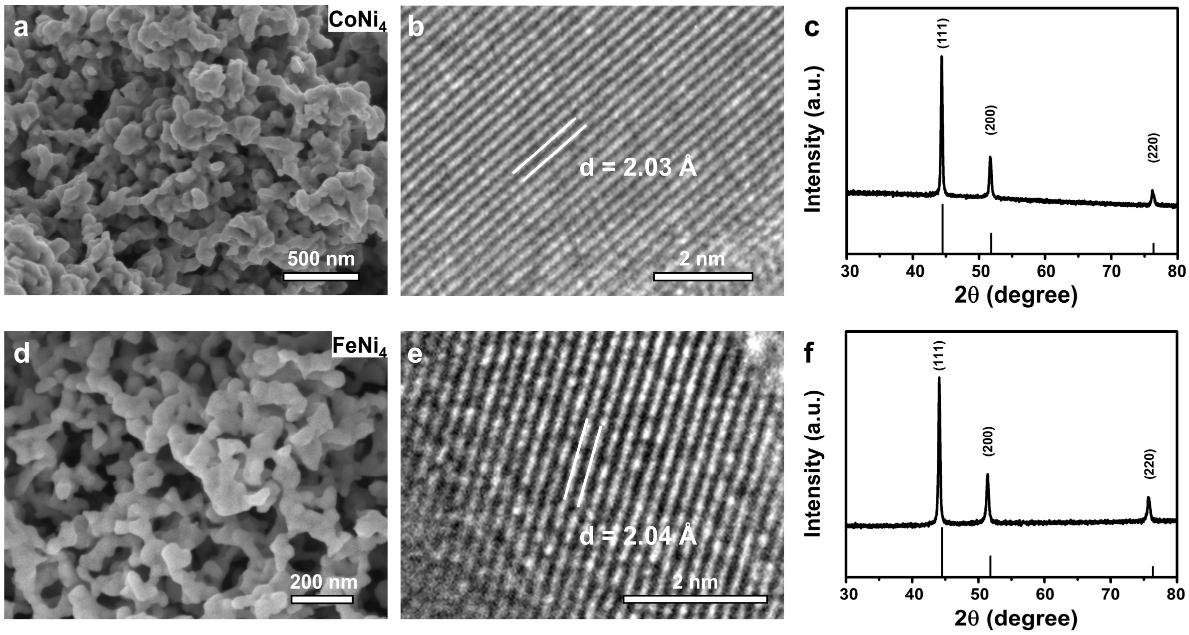

Supplementary Figure 44. Physical characterization of $CoNi_4$ and $FeNi_4$ alloys. a, **d,** SEM images of $CoNi_4$ and $FeNi_4$ alloys, respectively. **b, e,** HRTEM images of $CoNi_4$ and $FeNi_4$ alloys, respectively. **c, f,** XRD patterns of $CoNi_4$ and $FeNi_4$ alloys, respectively. The inserted lines in **c** and **f** are indexed to Ni (*JCPDS* 04-0850). The results demonstrate the well synthesis of $CoNi_4$ and $FeNi_4$ alloys.

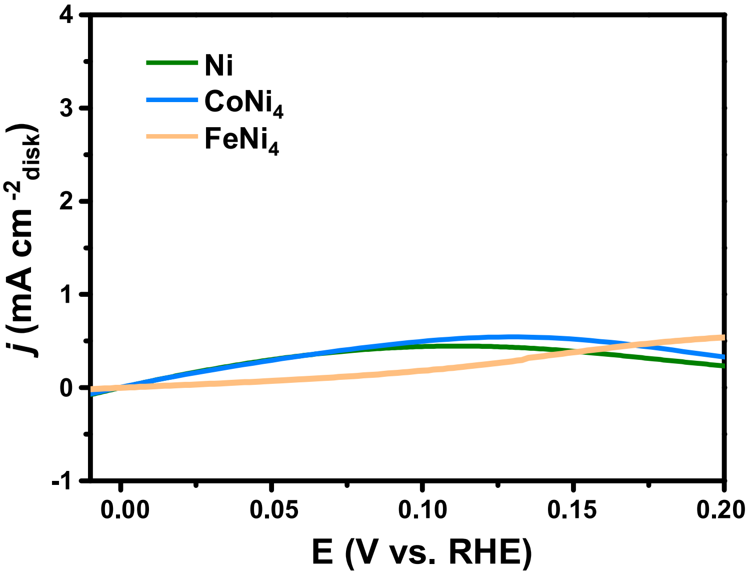

Supplementary Figure 45. HOR polarization curves of freshly-synthesized Ni, $CoNi_4$ and $FeNi_4$ alloys. It can been seen that $CoNi_4$ alloy shows slight activity enhancement compared with freshly-synthesized Ni. However, $FeNi_4$ alloy gives a decay signature. Based on DFT calculations, the HBE of $CoNi_4$ alloy shifts positively along with a negative shift of $FeNi_4$ alloy, which are consistent to the observed different HOR activities of freshly-synthesized Ni, $CoNi_4$ and $FeNi_4$ alloys.

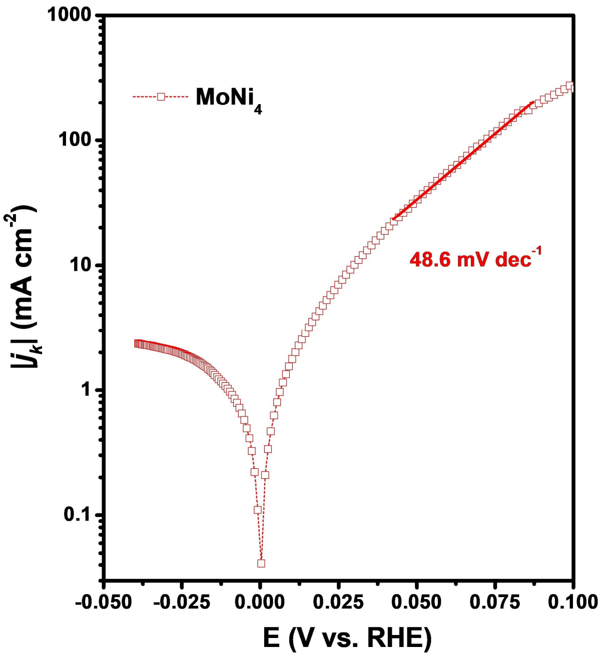

Supplementary Figure 46. HOR/HER Tafel plot of the kinetic current density on $MoNi_4$ in H_2-saturated 0.1 M KOH. The Tafel slope was obtained in the overpotential region from 50 to 100 mV to avoid the incorrect fitting in too low overpotential region based on the definition of the Tafel equation.

Supplementary Table 1. Atomic ratios of Ni-based alloys by ICP-AES results.

MNi_4 Alloys	M:Ni ratios
$MoNi_4$	1:3.94
WNi_4	1:3.92
$CoNi_4$	1:4.33
$FeNi_4$	1:3.57

Supplementary Table 2. EXAFS fitting parameters at the Ni K-edge and Mo K-edge for the studied samples.

Sample	Shell	N [a]	R (Å) [b]	σ^2 (Å $^2\cdot 10^{-3}$) [c]	ΔE_0 (eV) [d]	R factor (%)
$MoNi_4$	Ni-Ni(Mo)	8.8	2.49	7.9	4.6	0.1
WNi_4	Ni-Ni(W)	8.6	2.50	6.3	7.1	0.3
Ni	Ni-Ni	10.5	2.48	5.9	6.4	0.1
Ni foil	Ni-Ni	12	2.48	5.9	6.2	0.1
$MoNi_4$	Mo-Ni	6.9	2.52	6.1	-1.7	0.8

[a] *N*: coordination numbers; [b] *R*: bond distance; [c] σ^2: Debye-Waller factors; [d] ΔE_0: the inner potential correction. *R* factor: goodness of fit. S_0^2 for Ni-Ni was set as 0.83, which was obtained from the experimental EXAFS fit of Ni foil reference by fixing CN as the known crystallographic value and was fixed to all the samples.

Supplementary Table 3. Summary of the exchange current density of $MoNi_4$, WNi_4, freshly-synthesized Ni and Pt/C, which are calculated in micro-polarization regions and Tafel regions.

Catalysts	Exchange current density (mA cm^{-2}_{disk})	
	Micro polarization regions	Tafel regions
$MoNi_4$	3.41	3.23
WNi_4	1.87	1.73
Ni	0.19	0.15
Pt/C	2.47	2.26

Supplementary Table 4. Summary of the reported HOR catalytic performance of various PGM-free HOR catalysts measured in alkaline electrolyte (pH 13).

Catalysts	Rotating speed (rpm)	Kinetic current density@50 mV (mA cm^{-2}_{disk})	Exchange current density (mA cm^{-2}_{disk})	Exchange current density (mA cm^{-2}_{metal})	Reference
Ni/N-CNT	2500	2.33	0.89	0.028	14
Ni/CNT	2500	0.48	0.25	0.0092	14
Ni	2500	0.07	0.035	0.0013	14
NiCoMo	1600	-	-	0.015	7
NiMo/C	1600	-	~0.20	0.027	15
NiCu/C	1600	-	0.47	0.025	16
Ni_3N/C	2500	3.90	1.89	0.014	17
Ni_3N	2500	0.48	0.26	0.017	17
$Ni/CeO_2/C$	2500	1.73	1.07	0.038	18
Ni_3@BN/C	2500	-	0.84	0.023	19
$Ni/Ni_3N/NF$	-	-	-	0.003	20
Ni/NiO/C	1600	1.59	-	0.026	8
Cu	2500	-	-	0.0016	21
Co	2500	-	-	0.0032	21
Fe	2500	-	-	0.013	21
W	2500	-	-	6*E-5	21
Ni	1600	0.32	0.19	0.014	This Work
WNi_4	1600	8.31	1.87	0.068	This Work
$MoNi_4$	1600	33.8	3.41	0.065	This Work

Supplementary References

1 Mao, J. *et al.* Design of ultrathin Pt-Mo-Ni nanowire catalysts for ethanol electrooxidation. *Sci. Adv.* **3**, e1603068 (2017).

2 Yang, Y. *et al.* Versatile nickel–tungsten bimetallics/carbon nanofiber catalysts for direct conversion of cellulose to ethylene glycol. *Green Chem.* **18**, 3949-3955 (2016).

3 Zhang, J. *et al.* Efficient hydrogen production on $MoNi_4$ electrocatalysts with fast water dissociation kinetics. *Nat. Commun.* **8**, 15437 (2017).

4 Zhang, Q. *et al.* Superaerophobic ultrathin Ni–Mo alloy nanosheet array from in situ topotactic reduction for hydrogen evolution reaction. *Small* **13**, 1701648 (2017).

5 Nsanzimana, J. M. V. *et al.* An Earth-Abundant Tungsten–Nickel Alloy Electrocatalyst for Superior Hydrogen Evolution. *ACS Applied Nano Materials* **1**, 1228-1235 (2018).

6 Elias, L., Cao, P. & Hegde, A. C. Magnetoelectrodeposition of Ni–W alloy coatings for enhanced hydrogen evolution reaction. *RSC Advances* **6**, 111358-111365 (2016).

7 Sheng, W. *et al.* Non-precious metal electrocatalysts with high activity for hydrogen oxidation reaction in alkaline electrolytes. *Energy Environ. Sci.* **7**, 1719-1724 (2014).

8 Yang, Y. *et al.* Enhanced electrocatalytic hydrogen oxidation on Ni/NiO/C derived from a Ni-based MOF. *Angew. Chem. Int. Ed.* **58**, 10644-10649 (2019).

9 McKone, J. R., Sadtler, B. F., Werlang, C. A., Lewis, N. S. & Gray, H. B. Ni–Mo nanopowders for efficient electrochemical hydrogen evolution. *ACS Catal.* **3**, 166-169 (2013).

10 Xie, X., Chen, S., Ding, W., Nie, Y. & Wei, Z. An extraordinarily stable catalyst: Pt NPs supported on two-dimensional $Ti_3C_2X_2$ (X= OH, F) nanosheets for oxygen reduction reaction. *Chem. Commun.* **49**, 10112-10114 (2013).

11 Avasarala, B. & Haldar, P. Durability and degradation mechanism of titanium nitride based electrocatalysts for PEM (proton exchange membrane) fuel cell applications. *Energy* **57**, 545-553 (2013).

12 Jayaraman, S. & Hillier, A. C. Screening the reactivity of Pt_xRu_y and $Pt_xRu_yMo_z$ catalysts toward the hydrogen oxidation reaction with the scanning electrochemical microscope. *J. Phys. Chem. B* **107**, 5221-5230 (2003).

13 Chen, Z. *et al.* Tailoring the d-Band Centers Enables Co_4N Nanosheets To Be Highly Active for Hydrogen Evolution Catalysis. *Angew. Chem. Int. Ed.* **57**, 5076-5080 (2018).

14 Zhuang, Z. *et al*. Nickel supported on nitrogen-doped carbon nanotubes as hydrogen oxidation reaction catalyst in alkaline electrolyte. *Nat. Commun.* **7**, 10141 (2016).

15 Kabir, S. *et al*. Platinum group metal-free NiMo hydrogen oxidation catalysts: high performance and durability in alkaline exchange membrane fuel cells. *J. Mater. Chem.* A **5**, 24433-24443 (2017).

16 Roy, A. *et al*. Nickel–copper supported on a carbon black hydrogen oxidation catalyst integrated into an anion-exchange membrane fuel cell. *Sustain. Energ. Fuels* **2**, 2268-2275 (2018).

17 Ni, W. *et al*. Ni3N as an active hydrogen oxidation reaction catalyst in alkaline medium. *Angew. Chem. Int. Ed.* **58**, 7445-7449 (2019).

18 Yang, F. *et al*. Boosting Hydrogen Oxidation Activity of Ni in Alkaline Media through Oxygen‐Vacancy‐Rich CeO2/Ni Heterostructures. *Angew. Chem. Int. Ed.* **58**, 14179-14183 (2019).

19 Gao, L. *et al*. A nickel nanocatalyst within a h-BN shell for enhanced hydrogen oxidation reactions. *Chem. Sci.* **8**, 5728-5734 (2017).

20 Song, F. *et al*. Interfacing nickel nitride and nickel boosts both electrocatalytic hydrogen evolution and oxidation reactions. *Nat. Commun.* **9**, 4531 (2018).

21 Sheng, W., Myint, M., Chen, J. G. & Yan, Y. Correlating the hydrogen evolution reaction activity in alkaline electrolytes with the hydrogen binding energy on monometallic surfaces. *Energy Environ. Sci.* **6**, 1509-1512 (2013).
